# Eligibility or use? Disentangling the sources of horizontal inequity in home care receipt in the Netherlands

**DOI:** 10.1002/hec.4126

**Published:** 2020-07-08

**Authors:** Marianne Tenand, Pieter Bakx, Eddy van Doorslaer

**Affiliations:** ^1^ Erasmus School of Health Policy & Management (ESHPM) Erasmus University Rotterdam Rotterdam The Netherlands; ^2^ Erasmus School of Economics (ESE) Erasmus University Rotterdam Rotterdam The Netherlands

**Keywords:** eligibility, equity in care use, home care, horizontal equity, needs assessment, socioeconomic inequality

## Abstract

We study horizontal inequity in home care use in the Netherlands, where a social insurance scheme aims to allocate long‐term care according to care needs. Whether the system reaches its goal depends not only on whether eligible individuals have equal access to care but also on whether entitlements for care reflect needs, irrespective of socioeconomic status and other characteristics. We assess and decompose total inequity into inequity in (i) entitlements for home care and (ii) the conversion of these entitlements into actual use. This distinction is original and important, because inequity calls for different policy responses depending on the stage at which it arises. Linking survey and administrative data on the 65 and older, we find higher income elderly to receive less home care than poorer elderly with similar needs. Although lower income elderly tend to make greater use of their entitlements, need‐standardized entitlements are similar across income, education, and wealth levels. However, both use and entitlements vary by origin and place of residence. The Dutch need assessment seems effective at restricting socioeconomic inequity in home care use but may not fully prevent inequity along other dimensions. Low financial barriers and universal eligibility rules may help achieve equity in access but are not sufficient conditions.

## 1 INTRODUCTION

Limiting inequalities in access to long‐term care (LTC) seems a widely accepted policy objective, in particular in Europe where “the right to affordable long‐term care” is enshrined in the European Pillar of Social Rights (European Commission, 2017). Yet, in many countries, public coverage for LTC is limited, especially for those with moderate needs and for community‐based care provision (Muir, 2017); low‐income elderly are often exposed to a substantial risk of unmet needs, poverty, or involuntary reliance on informal care (OECD, 2017).

The Netherlands has put in place a comprehensive and generous LTC system that aims to ensure that LTC use is based only on care needs. We aim to answer the question whether this has ensured horizontal equity in home care use by Dutch elderly. The horizontal equity principle requires that people in equal need should receive equal care, irrespective of other irrelevant characteristics. Our focus is primarily on socioeconomic inequity: As LTC is extremely costly, ensuring that access to these services does not depend on the elderly's and their families' ability to pay is a primary policy concern in aging countries. Additionally, we document inequity in LTC access along with other sociodemographic factors as well as place of residence. The unfair access to care of some segments of the population is of policy interest per se; in addition, it can contribute to inequalities in LTC use by socioeconomic status (SES).

We combine administrative data and survey data from 2012 to decompose total horizontal inequity in home care use into two components: (1) inequity in the granting of entitlements for home care and (2) inequity in the conversion of these entitlements into actual use. This distinction is important because inequity remedies will be different depending on the stage at which it arises. Inequity in LTC use may not only be due to high out‐of‐pocket costs but also to the way in which access to benefits and provision of LTC are organized; thus, it may also occur in systems with very low‐cost or even entirely free care to users. In general, inequity in health care access may arise in each of three stages: (i) the decision to consult a health care provider for a certain health issue, (ii) the diagnosis and the prescription of a treatment by the provider, and (iii) the uptake or provision of the treatment. However, very often stages (ii) and (iii) cannot be assessed separately because the diagnosis, the choice of a treatment, and the provision of care are all decisions made by the same professional. In the case of LTC, however, in many countries, stage (ii) is organized as a separate eligibility assessment and delegated to agents who are not the care providers.

We focus on the Netherlands for three main reasons. First, the Netherlands is fairly unique in offering very comprehensive public LTC coverage and collecting extensive and precise data on eligibility and use of publicly funded LTC. As private LTC is virtually nonexistent, we capture most inequalities in home care access by focusing on administratively recorded home care use. Second, in 2012, the same needs assessment was followed for all LTC except for domestic help; this makes it particularly relevant to identify inequity arising at the stage of granting LTC entitlements, as any such inequity will have implications for equity in use for (almost) all home care and institutional care. Third, the institutional setting provides us with a measure of all LTC entitlements that can be used empirically to test whether (i) those with the same needs receive the same entitlements and (ii) those with the same entitlements use the same care. The Netherlands has entrusted needs assessments to a central independent agency rather than LTC providers, mainly to ensure an equitable assessment. However, it is difficult to a priori rule out disparities in entitlements between subpopulations. As there is no systematic screening of the elderly population, some population groups (e.g., low‐educated or isolated elderly) may be less likely to navigate the system, apply, and receive benefits. In addition, Bakx, Wouterse, van Doorslaer, and Wong (2018) have shown that some Dutch assessors are more lenient than others in their decision to entitle elderly applicants to a nursing home stay; differing degrees of leniency might lead to eligibility decisions being more favorable to some groups. ^1^However, the fact that cases are assigned to assessors randomly—within one of the 10 regional offices—limits the possibility that a certain type of applicants has a higher chance to be paired with a lenient assessor. Bakx et al. (2018) showed that there is no empirical correlation between the leniency of the assessor and the applicant's background characteristics collected in the application.


Most prior research has measured overall inequity in LTC use in one or more European countries, focusing either on socioeconomic inequity (Carrieri, Di Novi, & Orso, 2017; García‐Gómez, Hernández‐Quevedo, Jiménez‐Rubio, & Oliva‐Moreno, 2015; Ilinca, Rodrigues, & Schmidt, 2017; Rodrigues, Ilinca, & Schmidt, 2018) or on disparities across municipalities, in the context of the decentralized LTC provision of Nordic countries (e.g., Davey, Johansson, Malmberg, & Sundström, 2006). All of these studies rely on data that do not contain information on the entitlements for publicly subsidized LTC; thus, they could not shed any light on which stage generates inequity in LTC access. To our knowledge, there are three exceptions. Duell, Koolman and Portrait (2017) and Duell, Lindeboom, Koolman and Portrait (2019) investigated into regional disparities in access to nursing home care in the Netherlands. Duell et al. (2019) documented substantially larger practice variation in the conversion of entitlements for nursing home care into use. Also focusing on the stage of the conversion of entitlements into care use in the Netherlands, Tenand, Bakx and Van Doorslaer (2020) assessed socioeconomic inequity in LTC use, encompassing both home care and nursing home care. They concluded that poorer individuals tend to use more, or higher cost, LTC (more often nursing home care) than richer elderly, given similar needs. But these results will only translate in pro‐poor horizontal inequity in LTC use if there is no countervailing pro‐rich inequity in the other stages, that is, if entitlements for publicly subsidized LTC appropriately reflect the legitimate needs for LTC. Regarding nursing home care, Duell et al. (2017) reported that after standardizing on the case mix of applicants, the average probability that assessors grant entitlements ranges from 28% to 31% across the regional offices of the assessment agency—a fairly limited spread.

Like Duell et al. (2019), we separate inequality in entitlements from inequality in use. Yet we add to the understanding of the Dutch context and to the international literature in four important ways. First, we investigate into access to home care instead of nursing home care. Second, although Duell et al. (2019) specifically aimed at documenting regional disparities, we document horizontal inequity along multiple dimensions, focusing primarily on income‐related inequity. Third, our study population is the full (noninstitutionalized) elderly population; by contrast, Duell et al.'s (2019) study assessed inequality conditional on application (i.e., within the group of applicants). This implies that inequity caused by differences in the propensity to apply would not be picked up, although it is potentially an important driver of inequity even in wealthy countries (Hernanz, Malherbet, & Pellizzari, 2004). Finally, Duell et al. (2019) relied on information recorded in the application files by assessors; instead, we complement background information with rich survey information on health and functional status collected independently from the LTC eligibility assessment process, which we believe is critical to unveil potential unequal treatments of similar cases.

Our empirical approach identifies which factors are associated with lower access to home care, at each of the two stages, after systematic differences in needs have been controlled for using a regression‐based indirect standardization method (O'Donnell, van Doorslaer, & Wagstaff, 2012). Thus, we separate the contribution of need variables to inequality in home care from the contribution of potentially correlated illegitimate drivers of inequality.

We report four main findings. First, there is moderate pro‐poor socioeconomic horizontal inequity in home care use overall. Second, we find no evidence that eligibility assessment procedures systematically favor the elderly with higher income. Third, lower income elderly tend to use more home care for given needs primarily because they convert more of their home care entitlements into actual use. Fourth, we find evidence of horizontal inequity along other dimensions than income. In particular, even though the social insurance for LTC is a national scheme in the Netherlands, and in spite of the central agency's efforts to implement a uniform needs assessment procedure, substantial regional disparities are observed.

## 2 ELIGIBILITY FOR LONG‐TERM CARE AND USE OF CARE SERVICES IN THE NETHERLANDS

The Dutch public LTC insurance provides universal and fairly comprehensive coverage of LTC expenses. Until 2015, this was realized through a unified public LTC insurance program (AWBZ). ^2^Between 2013 and 2015, the Dutch LTC system went through a substantial reform (van Ginneken & Kroneman, 2015). We describe the system as it stood in 2012, as we assess the prereform situation. Eligibility for publicly financed LTC is determined by the Dutch LTC assessment agency (*Centrum Indicatiestelling Zorg*, CIZ), which is independent from the bodies funding and organizing public LTC provision. Although it is a centralized agency, CIZ has 10 regional offices. A needs assessment can be requested either by the person with care needs or by someone on their behalf, including a relative or a health care professional.

The assessor decides on the type and volume of care that the applicant is eligible for. Applicants with a more severe condition and a less supporting environment may become eligible for nursing home care (30% of applicants in 2012; Duell et al., 2017); others are eligible for home care, which encompasses nursing care, personal care, individual guidance, and group guidance.

Extensive guidelines from the Ministry of Health describe the information that may be collected and used by assessors (RMO, 2010). Decisions should be based on the functional limitations of the applicant, her health status, and a limited number of background characteristics. Those criteria exclude the applicant's income or wealth, but they include the presence of relatives: Able household members are expected to provide some personal care and assistance in the activities of daily living to their disabled relatives (Mot, 2010).

Beneficiaries most often receive in‐kind care, but they can also opt for vouchers to pay their own professional caregivers or informal caregivers. Elderly eligible for nursing home care may choose to stay at home and receive an equivalent package of home care services. The provision of care is organized at a regional level: 32 regional purchasing agencies (*zorgkantoren*) contract with providers.

The bulk of LTC is publicly financed, and within this system, only 8% of LTC expenditures (2012 level) are financed through cost‐sharing (Maarse & Jeurissen, 2016). ^3^The remainder is paid for by mandatory social security contributions and general government revenue (Schut, Sorbe, & Hoj, 2013) Copayments increase with income and vary by type of care. They are capped: The monthly fee is lower than €20 for beneficiaries with lowest incomes, whereas pocket allowances and various deductions ensure that the richest beneficiaries retain about 60% of their income after paying for nursing home costs (Muir, 2017). Financial barriers in LTC access are thus limited, and the needs assessment is not based on financial information.

Yet disparities in LTC access might still arise for a variety of reasons. First, some individuals may be less likely to apply for LTC benefits or less able to navigate the bureaucracy (e.g., low‐educated individuals and non‐Dutch speakers). Second, certain groups might be less willing or able to substitute informal care for formal home care (e.g., women vs. men, as the former are less likely to have a spouse alive when they experience functional limitations). Third, assessors could make decisions more favorable to some categories of the population. ^4^In sociology, empirical studies of decision‐making by street‐level bureaucrats have documented that background circumstances of applicants can considerably influence entitlements for social benefits (see, e.g., Scott, 1997). Furthermore, the “representative theory agency” posits that case workers will advocate the case of culturally similar patients more strongly (see, e.g., Meier and Bohte (2001)). The conversion of information into entitlements is still a human decision, not a standardized algorithm. Moreover, entitlements do not automatically translate into the receipt of home care services, which may be postponed or delayed by demand or supply factors. For example, some groups might have better information about care delivery, or they could receive priority from providers.

## 3 DATA AND STUDY SAMPLE

### 3.1 Survey information on health and functional status

We use the 2012 wave of the Dutch Health Monitor (*Gezondheidsmonitor*), a cross‐sectional, individual‐level survey. It collects self‐reported information on health status, chronic diseases, and depression, physical, cognitive, and sensory functional limitations as well as indicators of mobility restrictions. It thus provides a rich set of proxies for LTC needs. Survey weights make the sample representative of the population aged 65 and older living in a private household in January 2012.

### 3.2 Linked administrative data

We link the Health Monitor with administrative data from Statistics Netherlands available for the entire Dutch population, based on a unique pseudomyzed individual identifier. Four types of data are linked. First, we use data from CIZ on eligibility for publicly subsidized home care or nursing home care as well as on the use of in‐kind LTC services data from the Central Administration Office of the LTC insurance scheme (*Centraal Administratie Kantoor*, or CAK) and the take‐up of cash benefits from the health insurance information system (Vektis). Second, we link data from the Tax Office on household income and wealth for 2011. Third, we add sociodemographic information on migrant background (whether the elderly himself or herself, or a parent was born abroad), the number of children alive, and the municipality of residence taken from population registers (*Basisregistratie Personen*, or BRP). ^5^Reference day: October 15, 2012 (the midpoint of the survey collection period). Fourth, we link the 2011 claims data from mandatory health insurance and indicators of outpatient drug use for 84 categories of pharmaceuticals, according to the second‐level categories (pharmacological or therapeutic subgroup) of the Anatomical Therapeutic Chemical classification established by the World Health Organization, as of January 1, 2011.

### 3.3 Study population and sample

**FIGURE 1:**
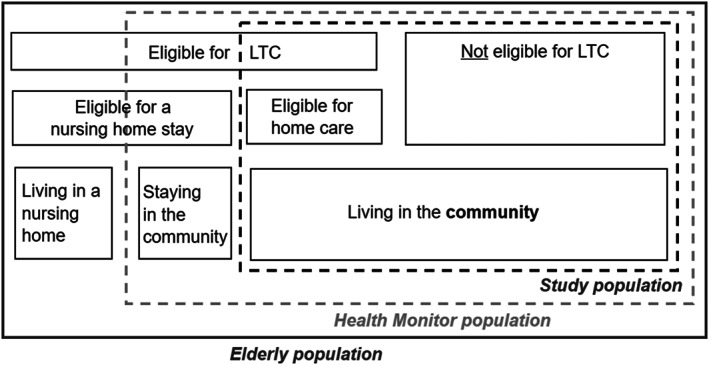
Definition of the study population

Starting from a sample of 164,988 individuals, representative of the noninstitutionalized 65 and older population, we select the study sample in two steps (Figure 1). First, we drop individuals who were eligible for nursing home care in 2012. This group includes both the elderly living in an LTC institution at the time of the survey (September–November 2012), ^6^Although the survey targeted individuals living in the community as of January 1, 2012, some respondents had entered an institution by the time of the survey and filled in the questionnaire. There are 1,550 such respondents aged 65 or older. Furthermore, we discard the few individuals who were found to be in any other type of institutional setting (e.g., mental health care facility or center for the handicapped) in the administrative data. as well as 3,607 respondents (2.1% of initial sample) who decided not to take up institutional care (yet) although they were eligible for it. This prevents the sample from being selected on the basis of respondents' decision to use certain LTC services. This first step delimits our study population, as depicted in Figure 1, and explains the focus on home care. Second, we drop individuals with missing information for any of the linked administrative variables (5,122 individuals). The final sample consists of 154,709 individuals (93.7% of initial sample).

## 4 METHODS

### 4.1 Three nested analyses

We run three nested analyses, described in Table 1. In Analysis A, we assess overall socioeconomic horizontal inequity in home care use as inequality in home care use across different population groups (in particular, across SES) after controlling for differences in home care needs. In Analysis B, we focus on horizontal equity in entitlements for home care, by assessing whether individuals with similar needs receive similar entitlements. Finally, Analysis C focuses on individuals eligible for home care and measures inequalities in the conversion of entitlements into actual use differs.

**TABLE 1:** The three stages of the empirical analysis

Analysis	Outcome	Sample	Standardized outcome
Analysis A: Horizontal Inequity (HI) in home care use	Value of home care use	Entire sample *N* = 154,709	Outcome standardized on needs. Derived from a regression of the outcome on need and nonneed factors (cf. Section 4.3)
Analysis B: HI in home care entitlements	Value of home care entitlements		
Analysis C: HI in the conversion of entitlements into home care use	Value of home care use	Sample eligible for home care *N* = 14,138	Care use standardized on entitlements (cf. Section 4.3)

### 4.2 Definition of LTC use and entitlements

Home care entitlements are computed as the monetary value of home care services covered by the LTC social insurance (AWBZ) that the individual was entitled to receive for a somatic or a psychogeriatric condition. The definition of home care does not include domestic help, which is financed through a separate scheme (Wmo). Home care entitlements are defined as a range of hours per week. We take the midpoint of the range when computing the values of entitlements and use.

Home care use is defined as the monetary value of all in‐kind home care services, plus the imputed value of LTC vouchers, that the individual received in 2012. For in‐kind services, we multiply quantities used by the price cap set by the Dutch Healthcare Authority (NZA). If individuals opt for vouchers rather than in‐kind care, we only observe whether they take up the vouchers—not the amount spent. In this case, we impute the monetary value of home care received by exploiting the official matrix used to convert home care entitlements into vouchers. ^7^The cash equivalent of in‐kind services represents about 75% of the national tariff of these services (see Tenand et al., 2020). Beneficiaries use on average 89.5% of the value of vouchers granted (Statistics Netherlands, 2018). Thus, the value of a voucher for an individual is imputed as 89.5% times 75% times the monetary value of the equivalent in‐kind care.


### 4.3 Need‐standardized outcomes

The egalitarian principle of horizontal equity requires that people with equal needs are treated equally by the LTC system. We measure the degree of inequity as the degree of inequality that remains after controlling for differences in the factors considered to legitimate greater LTC use. We use an indirect standardization method (O'Donnell et al., 2012).

We start by determining the need‐predicted outcomes. For each individual *i*, ${\hat{U}}_{i}^{A}$ and ${\hat{E}}_{i}^{B}$ correspond to the values of home care use and entitlements that would have been observed if home care use and entitlements only reflected care needs. In order to derive ${\hat{U}}_{i}^{A}$ and ${\hat{E}}_{i}^{B}$, we first estimate the following equations using Ordinary Least Squares (OLS):

(1A) $$U_{i}=\alpha_{0}+X_{i}^{\prime }\alpha^{X}+Z_{i}^{\prime }\alpha^{Z}+u_{i},$$

(1B) $$E_{i}=\beta_{0}+X_{i}^{\prime }\beta^{X}+Z_{i}^{\prime }\beta^{Z}+\varepsilon_{i},$$

where *U*_*i*_ is the home care use; *E*_*i*_ indicates the home care entitlements; *X*_*i*_ is the vector of care need factors; *Z*_*i*_ is the vector of other observable determinants of home care use, called nonneed factors; and *u*_*i*_ and *ε*_*i*_ are the error terms.

We use the estimates from Equations 1A and 1B to construct need‐predicted outcomes as follows:

(2A) $${\hat{U}}_{i}^{A}={\hat{\alpha }}_{0}+X_{i}^{\prime }{\hat{\alpha }}^{X}+{\bar{Z}}^{\prime }{\hat{\alpha }}^{Z},$$

(2B) $${\hat{E}}_{i}^{B}={\hat{\beta }}_{0}+X_{i}^{\prime }{\hat{\beta }}^{X}+{\bar{Z}}^{\prime }{\hat{\beta }}^{Z},$$

where $\bar{Z}$ is the vector of nonneed factors averaged over the study population. ^8^Population averages are computed using the survey weights.


Finally, we compute need‐standardized use ${\tilde{U}}_{i}^{A}$ and entitlements ${\tilde{E}}_{i}^{B}$ as follows:

(3A) $${\tilde{U}}_{i}^{A}=U_{i}-{\hat{U}}_{i}^{A}+\bar{U},$$

(3B) $${\tilde{E}}_{i}^{B}=E_{i}-{\hat{E}}_{i}^{B}+\bar{E}.$$

where $\overset{‐}{U}$ and $\overset{‐}{E}$ are the population average of care use and entitlements respectively. Interindividual differences in need‐standardized use and in need‐standardized entitlements reflect the differences that cannot be explained by observed differences in needs; as such, they provide a measure of horizontal inequity or illegitimate inequality.

For Analysis C, we derive the value of home care use standardized for LTC entitlements, ${\tilde{U}}_{i}^{C}$, so as to capture the interindividual variations in use that cannot be explained by individuals having different levels of home care entitlements.

(3C) $${\tilde{U}}_{i}^{C}=U_{i}-\left(\frac{{\bar{U}}^{\text{elig}}}{{\bar{E}}^{\text{elig}}}\right)E_{i}+{\bar{U}}^{\text{elig}},$$

where ${\bar{U}}^{\text{elig}}$ and ${\bar{E}}^{\text{elig}}$ are respectively home care use and entitlements averaged over the subpopulation eligible for home care. With this standardization, we measure horizontal inequity in relative terms, as the deviation from a situation in which all individuals with the same needs have the same home care use (“equal care for equal needs”) but in which average care use can possibly differ from average entitlements. Horizontal equity in care use is achieved whenever: $U_{i}=\left({\bar{U}}^{\text{elig}}/{\bar{E}}^{\text{elig}}\right)E_{i}$ for all individuals, even if ${\bar{U}}^{\text{elig}}/{\bar{E}}^{\text{elig}}$ is significantly below 1 (as it is the case here; cf. Section 4). Note that contrary to Analyses A and B, the standardized outcome of Analysis C is not regression based: In the latter case, we observe entitlements, whereas in the former case, we statistically derive a measure of needs.

### 4.4 Concentration index and horizontal inequity index

This paper focuses primarily on socioeconomic inequity in LTC access. We use the horizontal inequity index as a synthetic measure of this dimension of inequity (van Doorslaer & Van Ourti, 2011) for each of Analyses A, B, and C. Formally, indices *HI*^*A*^, *HI*^*B*^, and *HI*^*C*^ are equal to the following:

(4A) $${\mathrm{HI}}^{A}=\mathrm{CI}\left(U\right)-\mathrm{CI}\left({\hat{U}}^{A}\right)=\mathrm{CI}\left({\tilde{U}}^{A}\right),$$

(4B) $${\mathrm{HI}}^{B}=\mathrm{CI}\left(E\right)-\mathrm{CI}\left({\hat{E}}^{B}\right)=\mathrm{CI}\left({\tilde{E}}^{B}\right),$$

(4C) $${\mathrm{HI}}^{C}=\mathrm{CI}{\left(U\right)}^{\text{elig}}-\mathrm{CI}{\left(E\right)}^{\text{elig}}=\mathrm{CI}\left({\tilde{U}}^{C}\right),$$

with the concentration index over SES of a continuous variable *Y*, *CI*(*Y*), computed as follows:

(5) $$\mathrm{CI}\left(Y\right)=\frac{2\mathrm{cov}\left(Y,r^{\mathrm{SES}}\right)}{\bar{Y}},$$

where $r_{i}^{\mathrm{SES}}$ is the weighted fractional rank of individual *i* in the distribution of SES and $\bar{Y}$ is the population average of *Y*. ^9^For the individual ranking *i*th in the distribution from lowest to highest SES, $r_{i}^{\mathrm{SES}}=\sum_{j=0}^{i-1}w_{j}+\left(w_{i}/2\right)$, where *w*_*j*_ is the sample weight (rescaled to sum to 1) and *w*_0_ = 0 (O'Donnell, van Doorslaer, Wagstaff, & Lindelow, 2008).
^10^The difference between *CI*(*U*) in Equation 4A and *CI*(*U*)^*elig*^ in Equation 4B is that the former measures the concentration of home care use in the full study population, whereas the latter measures the concentration of home care use among the elderly eligible for home care. A similar distinction applies for the difference between *CI*(*E*) in Equation 4B and *CI*(*E*)^*elig*^ in Equation 4C. The concentration index varies from −1 (maximum pro‐poor inequality in the distribution of the outcome) to +1 (maximum pro‐rich inequality). If it is equal to 0, outcome *Y* is equally distributed across income levels on balance (Wagstaff & van Doorslaer, 2000).

### 4.5 Income as indicator of SES

We proxy SES by disposable household income adjusted for household size ^11^We use the OECD square root equivalence scale (OECD, 2011). for three main reasons. First, income is a continuous ranking variable, unlike, for example, education. Second, income includes the flow of pension and other benefits—a major economic resource for the elderly—whereas administrative data on wealth do not include Social Security wealth. We therefore believe that ranking the elderly based on their income better captures the potential importance of economic resources in LTC access. Third, income is the most common variable used by policymakers to monitor SES disparities in the access to public services and social benefits.

### 4.6 Horizontal inequity along other dimensions than income

In the empirical analysis, we additionally test for systematic differences in average standardized use or entitlements across population groups (e.g., males vs. females, or the low educated vs. the high educated) in order to assess horizontal inequity along dimensions other than income.

### 4.7 Classification of need and nonneed factors

The findings from Analyses A and B, in terms of inequity along with income and other dimensions, will critically depend on which variables are included in Equations 1A and 1B. Their selection is based on two criteria.

First, we follow the perspective of the Ministry of Health and of CIZ to define the legitimate sources of inequalities in LTC use (CIZ, 2012). We use survey measures of health, chronic conditions, functional limitations, depression, and loneliness. Additional need variables include health care costs and drug use in the previous year. Since in the Netherlands household members are expected to provide some informal care to a disabled relative, we include a dummy for the presence of a partner as a need‐reducing factor. As an exception, we also include age as a need variable even though it is not a criterion for access to (more) home care: We posit that age predominantly captures a condition of frailty that is not entirely reflected by self‐reported health and health care use (Sirven & Rapp, 2017).

Given the large number of need variables identified, we run the risk of overfitting the model when estimating Equations 1A and 1B and of obtaining imprecise estimates of need‐predicted outcomes (cf. Equations 2A and 2B). In order to narrow down the list of need variables, we implement a Lasso procedure ^12^Lasso (least absolute shrinkage and selection operator) is a regression‐based statistical method that can be used for variable selection and to enhance prediction accuracy. (described in Appendix A, Data S1) instead of hand‐picking a subset of variables.

Second, nonneed factors encompass all illegitimate determinants of home care entitlements or use that may correlate with needs. Failing to include such variables may result in biased estimates of *α*^*X*^ and *β*^*X*^ (Gravelle, 2003), which will in turn bias need‐standardized outcomes. ^13^Our methodology thus differs from Duell et al. (2019) who, following the practice variation literature, do not take into account nonneed factors when standardizing use and entitlements on need variables. Therefore, instead of shrinking down the number of nonneed factors, we include the richest possible set of such variables: gender, per capita household wealth (in deciles), home ownership, migrant background, education, size of city of residence, the CIZ regional office, and the LTC purchasing region the respondent lives in. ^14^We group these 32 regions into 8 groups, to avoid technical issues when performing Bootstrap inference (see Online Appendix C). As family characteristics beyond household composition are not supposed to be taken into account by CIZ, the number of children, the number of daughters, and the geographical proximity of children are part of the list of nonneed factors.

## 5 RESULTS

### 5.1 Descriptive statistics

Summary statistics of the study population are provided in Table 2, in Column (1) for the entire study population and in Column (2) for the 9.7% individuals who were eligible for home care at some point in 2012. In the entire population, the prevalence of mobility and sensory limitations, other functional limitations, and self‐reported chronic conditions is high, which is in line with the high average age (73.7 years). Poor health and functional limitations are more prevalent in the subpopulation entitled to home care, which also tends to be older and more often single. These statistics confirm that home care entitlements are, on average, targeted toward the elderly with higher needs. Elderly eligible for home care are also more likely to be women, to have children living in their municipality of residence, and to have lower education, wealth, and income.

**TABLE 2:** Summary descriptive statistics

		Entire weighted sample	Eligible for home care
		(1)	(2)
A. Demographic characteristics			
	Women	53.6%	66.5%
	Age	73.7	80.0
	Deceased in 2012	0.2%	1.4%
	Of Dutch descent	86.1%	84.8%
	Spouse alive	63.0%	37.0%
	Number of children alive	2.1	2.3
	No child	13.2%	15.1%
	Closest child lives at the same address	4.9%	5.0%
	Closest child lives in the same city (but different address)	51.9%	55.0%
	Closest child lives in a different city	30.0%	25.0%
	Lives in a municipality with less than 10,000 inhabitants	1.6%	1.2%
	Lives in a city with 10,001 to 50,000 inhabitants	50.9%	50.0%
	Lives in a city with 50,001 to 150,000 inhabitants	29.2%	28.9%
	Lives in a city with more than 150,000 inhabitants	18.4%	19.9%
B. Socioeconomic status			
	Education level: elementary	60.8%	71.2%
	Education level: secondary or unknown	21.9%	20.3%
	Education level: tertiary	17.3%	8.5%
	Disposable income	€30,780	€24,608
	Wealth (per capita)	€162,333	€128,343
C. LTC eligibility and use in 2012			
	Eligible for home care (yes/no)	9.7%	100.0%
	Entitlements to home care (value) if entitled	€14,788	€14,788
	Home care (in kind) (yes/no)	8.1%	83.4%
	Personal budget (yes/no)	0.4%	4.6%
	LTC use (value) if using	€8,690	€8,690
D. Functional limitations			
	Any functional limitation	59.2%	87.3%
	(a) Difficulties to follow a conversation	32.2%	51.1%
	(b) Difficulties to get involved into a conversation	11.3%	25.4%
	(c) Can read small characters	23.0%	42.1%
	(d) Difficulties to recognize faces in the street	12.0%	27.0%
	(e) Difficulties to lift shopping bags	30.9%	73.1%
	(f) Difficulties to bend and lift some weight	28.9%	75.0%
	(g) Difficulties to walk short distances	28.6%	66.8%
	Any mobility or sensory limitations	28.1%	68.1%
	(a) Hearing limitations	7.6%	18.7%
	(b) Sight limitations	8.1%	20.3%
	(c) Mobility limitations	21.1%	62.3%
E. Self‐assessed health (SAH)			
	SAH: good	60.8%	25.4%
	SAH: average	34.0%	54.7%
	SAH: bad	5.2%	19.9%
	Has a chronic condition	79.1%	89.3%
	Number of chronic conditions, conditional on having any	2.4	3.9
	Feels lonely (moderately to seriously)	44.3%	71.5%
	Feels anxious/depressed	37.1%	65.0%
F. Health care costs and drug use in 2011			
	Total health care costs (covered by mandatory insurance)	€3,586	€8,026
	General Practitioner care costs	194€	€288
	Any pharmaceutical use	5.1%	12.5%
	Population size (estimated)	2,600,000	297,000
	Sample size	154,709	14,138

*Note*: Values of eligibility, use, and income are expressed in euros per year. Figures are weighted by survey weights.

Abbreviation: LTC, long‐term care.

### 5.2 Income‐related horizontal inequity

In terms of descriptive statistics, although 16% of the elderly in the bottom income decile are entitled to home care in 2012, this figure is less than 4% in the top decile. The income‐gradient in care use is almost equally marked: 14% of the 10% poorest elderly receive in‐kind home care or or LTC vouchers, but only 3% of the top decile.

The estimates of the concentration indices and horizontal inequity indices in Table 3 confirm a strong pro‐poor concentration of home care use (−0.341 in the entire population). But the value of home care entitlements also exhibits a highly negative concentration index (−0.288): Entitlements are on balance higher for lower income elderly and also among those entitled to home care (−0.041). ^15^That *CI*(*U*) is smaller in Analysis C than in Analysis A is expected, as only a minority of the subpopulation eligible for home care has zero use. Same remark applies for *CI*(*E*) in Analysis B compared with Analysis C.


**TABLE 3:** CI and HI for Analyses A, B, and C

	CI (outcome)	CI (need‐predicted outcome)	HI (outcome)	N
Analysis	(1)	(2)	(3) = (1) − (2)	
A. Home care use (equity overall)	−0.340^***^ [−0.361, −0.315]	−0.304^***^ [−0.318, −0.292]	−0.036^***^ [−0.057, −0.010]	154,709
B. Home care entitlements (equity at eligibility stage)	−0.288^***^ [−0.311, −0.267]	−0.272^***^ [−0.286, −0.261]	−0.016^n.s.^ [−0.036, −0.005]	154,709
C. Conversion of entitlements into use (equity at use stage)	−0.095^***^ [−0.113, −0.071]	−0.040^***^ [−0.056, −0.022]	−0.054^***^ [−0.065, −0.040]	14,138

*Note*: The figures in brackets show asymmetric 95% confidence intervals, computed using a bootstrap approach (1,000 replications; see Appendix C, Data S1).

Abbreviations: CI, concentration index; HI, horizontal inequity index.

^***^ *p* < 0.01.

^**^ *p* < 0.05.

^*^ *p* < 0.10.

^n.s.^ *p* ≥ 0.10.

Analysis A shows that the pro‐poor concentration of needs (−0.305) does not entirely offset the unequal distribution of home care use: This results in a negative HI (−0.036). HI for Analysis B is also negative (−0.015) but not statistically significantly different from 0. Our bootstrapped 95% confidence interval suggests that there is a 95% chance that HI is lower than 0.005: Our findings reject the hypothesis of pro‐rich inequity at the stage of home care eligibility. Pro‐poor horizontal inequity in home care use is thus not due to poorer elderly receiving greater home care entitlements.

From Analysis C, it seems that inequity in home care use stems mainly from the conversion of entitlements into actual use: Among those eligible for home care, the pro‐poor distribution of entitlements (−0.041) does not suffice to explain the pro‐poor distribution of LTC use: HI is markedly negative (−0.054).

**FIGURE 2:**
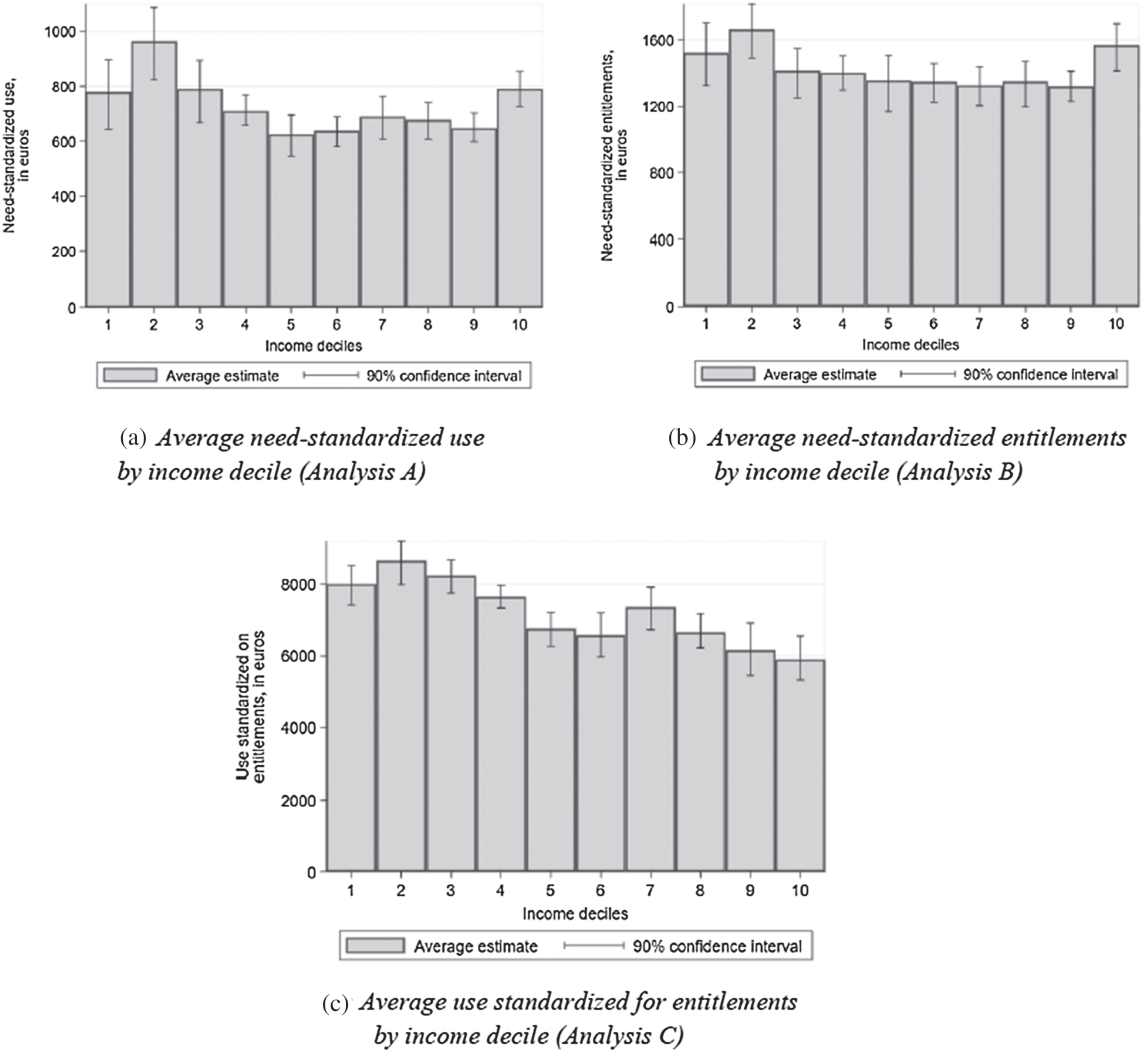
Average standardized outcomes, by income decile (Analyses A, B, and C). (a) Average need‐standardized use by income decile (Analysis A). (b) Average need‐standardized entitlements by income decile (Analysis B). (c) Average use standardized for entitlements by income decile (Analysis C). Samples: Weighted study sample (Panels a and b); weighted subsample eligible for home care (Panel c). Notes: Need‐standardized use and entitlements (Analyses A and B) and use standardized for entitlements (Analysis C) are defined in Equations 3A to 3C. Asymmetric 90% level confidence interval based on 1,000 Bootstrap replications (see Appendix C, Data S1). Values are expressed in euros over 2012

To ease interpretation of the magnitude of income‐related horizontal inequity, Figure 2 compares average standardized outcomes across income deciles. Figure 2a reveals that the 30% poorest elderly use on average 27% more home care (in value) than the elderly in the middle‐top of the income distribution (deciles 4 to 9), when needs are controlled for. The 10% richest elderly, however, show somewhat higher use. The close‐to‐zero HI of Analysis B is the result of care entitlements being similar for deciles 3 to 9, whereas the poorest (deciles 1 and 2) but also the richest (decile 10) receive 17% higher entitlements, for given needs (Figure 2b). Instead, at the stage of the conversion of entitlements into use, Figure 2c shows a roughly monotonous and markedly negative income gradient. The eligible elderly among the 10% poorest use 36% more home care than the elderly in the top decile, for given entitlements. In other words, the poorest convert one third more of their entitlements into use than the richest.

### 5.3 Further dimensions of horizontal inequity in home care access

**FIGURE 3:**
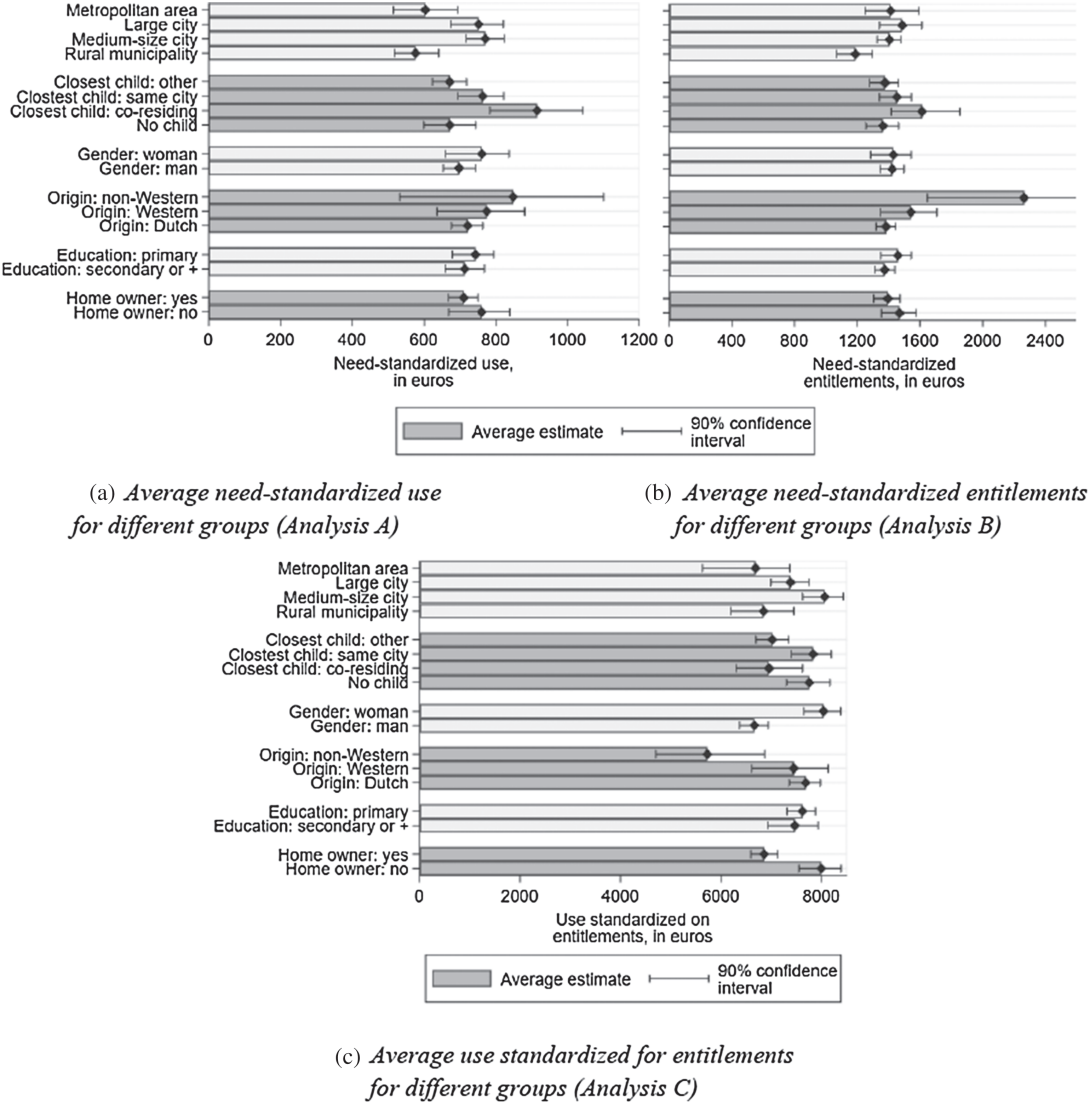
Average standardized outcomes, by different groups (Analyses A, B, and C). (a) Average need‐standardized use for different groups (Analysis A). (b) Average need‐standardized entitlements for different groups (Analysis B). (c) Average use standardized for entitlements for different groups (Analysis C). Samples: Weighted study sample (Panels a and b); weighted subsample eligible for home care (Panel c). Notes: Need‐standardized use and entitlements (Analyses A and B) and use standardized for entitlements (Analysis C) are defined in Equations 3A to 3C. Asymmetric 90% level confidence interval based on 1,000 Bootstrap replications (see Appendix C, Data S). Values are expressed in euros over 2012

We now assess several other dimensions of horizontal inequity in home care use, again decomposing into inequity arising at the stage of eligibility and inequity at the stage of entitlements conversion. Figure 3 displays need‐standardized home care use (Figure 3a) and need‐standardized entitlements (Figure 3b) averaged within the study population among subgroups defined by nonneed characteristics; Figure 3c presents home care use averaged over the same groups but within the subpopulation eligible for home care. To save space, standardized outcomes across wealth deciles and regions are reported in Figures B.1 to B.3 in Appendix B, Data S1.

What can explain why a population group has lower home care use, after controlling for its needs? There are two potential channels: (i) Belonging to this group (e.g., having a low education level) is per se associated with lower use when other nonneed factors are controlled for, on top of care needs; or (ii) the composition of this group (e.g., the low educated) in terms of socioeconomic, demographic, and geographic characteristics leads to its lower use of care. The coefficient estimates of Equations 1A and 1B reflect the partial correlation between each of the nonneed factors of either home care use or entitlements when controlling for need variablesand the other nonneed factors. As such, they help to understand the channels underlying horizontal inequity; they are reported in Columns (1) and (3) of Table 4. ^16^Suppose that we read from Figure 3 that need‐standardized use is lower for group X than for group Z. If the coefficient of the dummy for group X in Table B.III (Column 1) in Appendix B, Data S1 were not statistically nor practically significant, we would conclude that horizontal inequity between groups X and Y stems from the socioeconomic and demographic composition of the two groups beyond their difference in education levels. Similarly, for Analysis C, Column (5) of Table 4 provides the partial correlation between each of the nonneed factors and home care use, when controlling for entitlements and other nonneed factors.

**TABLE 4:** Regression estimates for nonneed factors—Analyses A, B, and C

	Analysis A		Analysis B		Analysis C	
Outcome	LTC use (value)		LTC entitlements (value)		LTC use (value)	
	Coef.	SE	Coef.	SE	Coef.	SE
	(1)	(2)	(3)	(4)	(5)	(6)
Need variables	Yes		Yes		No	
Home care entitlements	No		No		0.512	‐
Nonneed factors						
Income decile: 1	147.8^*^	80.5	45.5	124.3	1354.2^**^	618.3
Income decile: 2	306.5^***^	101.1	237.6	183.6	1662.7^***^	474.6
Income decile: 3	136.8	100.5	15.0	179.2	1128.2^***^	369.8
Income decile: 4	70.4	77.3	26.7	169.4	635.0	385.7
Income decile: 5	*Ref.*	*‐*	*Ref.*	*‐*	*Ref.*	*‐*
Income decile: 6	21.1	52.4	11.2	126.9	−436.4	632.3
Income decile: 7	76.3	54.8	−3.7	110.9	451.4	602.7
Income decile: 8	79.7	53.4	37.6	83.0	−300.3	390.0
Income decile: 9	57.4	58.3	23.2	135.8	−869.9	605.1
Income decile: 10	242.0^***^	82.3	300.7^*^	162.5	−749.6	524.0
Wealth decile: 1	−347.1^***^	100.7	−271.2^*^	152.3	−1973.7^***^	512.8
Wealth decile: 2	−85.5	110.6	−188.1	168.4	−248.9	423.5
Wealth decile: 3	−52.6	100.0	−35.2	149.0	−796.8	496.7
Wealth decile: 4	128.9	160.9	−11.0	188.0	655.5	587.0
Wealth decile: 5	*Ref.*	*‐*	*Ref.*	*‐*	*Ref.*	*‐*
Wealth decile: 6	−25.7	67.6	72.7	142.7	−1353.9^***^	475.8
Wealth decile: 7	81.5	88.0	130.5	145.2	247.8	475.5
Wealth decile: 8	−1.6	60.5	10.5	103.3	294.8	469.2
Wealth decile: 9	9.4	58.5	43.6	98.3	591.4	489.1
Wealth decile: 10	−5.2	68.7	193.4	129.6	−143.3	628.2
Homeowner	−157.4	82.7	−243.2^**^	101.0	−1561.3^***^	416.1
Female	45.6	79.8	72.7	142.7	1073.0^***^	247.5
Origin: Dutch	*Ref.*	*‐*	*Ref.*	*‐*	*Ref.*	*‐*
Origin: Western country, Dutch Indies	74.1	94.6	167.5	122.9	−219.0	573.8
Origin: non‐Western country	264.5	164.5	940.7^**^	422.0	−1548.9^**^	648.4
Education: primary	−2.3	54.0	86.0	68.0	−900.3^*^	516.4
Education: secondary	8.9	45.6	−24.5	82.9	−182.6	453.5
Education: tertiary	*Ref.*	*‐*	*Ref*	*‐*	*Ref.*	*‐*
Education: unknown	165.5	210.9	172.3	201.8	220.9	869.7
Number of children	41.5^*^	24.7	113.9^**^	45.5	−129.1	100.8
Number of daughters	8.6	25.4	19.0	50.8	34.8	139.8
No child	−116.6	98.8	160.1	219.0	−1445.0^***^	510.8
Closest child: at the same address	*Ref.*	*‐*	*Ref.*	*‐*	*Ref.*	*‐*
Closest child: same municipality	−155.0	101.9	−55.6	206.8	−1084.1^**^	487.7
Closest child: different municipality	−209.7^**^	82.8	−36.9	215.3	−1753.4^***^	541.8
Municipality of residence: small city/rural	−109.9^**^	50.8	−191.2^*^	111.0	−365.2	497.1
Municipality of residence: medium city	−24.3	37.2	−142.4	91.5	493.3	349.9
Municipality of residence: large city	*Ref.*	*‐*	*Ref.*	*‐*	*Ref.*	*‐*
Municipality of residence: metropole	−74.8	52.7	26.5	96.7	−543.6	500.3
Dummies for CIZ regional office	Yes	*p* = 0.000	Yes	*p* = 0.000	Yes	*p* = 0.006
Dummies for group of purchasing regions	Yes	*p* = 0.000	Yes	*p* = 0.000	Yes	*p* = 0.105
Constant	−281.8	260.4	−23.7	560.9	No	‐
*N*	154,646		154,646		14,136	
*R* ^2^	0.158		0.166		‐	

*Note*: Outcomes are expressed in euros over year 2012. Weighted linear estimations of Equations 1A for Columns (1) and (2) and 1B for Columns (3) and (4) (defined in Section 3). Columns (5) and (6) show the estimates of a weighted linear regression of home care use on entitlements and nonneed factors. The estimations take into account the clustered design of the sample (335 primary sampling units). When performing the estimations, a few observations for which the primary sampling unit is missing have to be dropped. The *p* values of a Fisher's test for joint statistical significance of the dummies are for CIZ regional offices and for LTC purchasing regions, respectively. Estimates of the need variables of Analyses A and B are reported in Table B.I in Appendix B, Data S1. For Analysis C, a constrained linear MLE is performed: The coefficient of entitlements is constrained to the value ${\bar{U}}^{\text{elig}}/{\bar{E}}^{\text{elig}}$ (see Equation 1.C in Appendix D.2, Data S1).

^***^ *p* < 0.01.

^**^ *p* < 0.05.

^*^ *p* < 0.10.

^n.s.^ *p* ≥ 0.10.

Starting with socioeconomic characteristics, Figure 3c reveals that homeowners use 12% less home care for given entitlements, possibly because they can more easily substitute home adaptation for human assistance than renters. Differences in entitlements across education levels are limited; however, having a primary education only is associated with sizably less home care use, for all other nonneed factors equal and given entitlements (Table 4, Column 5). Conditional on needs, elderly with little wealth do not appear to be at a systematic (dis)advantage in terms of home care use or entitlements. However, when further conditioning on other nonneed factors, being among the 10% elderly with the lowest wealth is associated with lower entitlements and use (Table 4, Columns 1 and 3).

Those with a non‐Western migrant background receive 60% more home care entitlements for given needs (Figure 3b). However, for given entitlements, they use 25% less care (Figure 3c). This explains that the elderly with a non‐Western migrant background have slightly higher use for given needs (Figure 3a), although the difference with the other elderly is not statistically significant at the 10% level. The gap in entitlements, which persists when we control for socioeconomic, demographic, and geographical characteristics, possibly occurs because CIZ assessors consider the (to us unobserved) social circumstances of migrant elderly to be less supportive because of weaker ties with the local community and public services. Other potential reasons why our analysis might underestimate care needs for the elderly with a migrant background is that self‐reported indicators of functional status may be sensitive to cultural differences or that language barriers might hamper contacts with physicians. The lower use of home care may also stem from cultural differences with respect to care use or in the propensity to call on substitute care from relatives.

Elderly with a coresiding child receive more home care for given needs than those with no child or a child living outside the household (Figure 3a). Other things equal, they convert more of their entitlements into care use (Table 4, Column 5). Given that these elderly are more likely to receive (daily) informal care than the elderly without children, or with children who live further away, this result runs counter to the hypothesis that having a coresiding child fosters the substitution of informal care for formal care. It is however in line with informal caregivers playing a key role in the activation of entitlements, by performing tasks complementary to the ones done by professional caregivers (Bonsang, 2009) and facilitating coordination among providers.

Women receive 20% more home care than males for given entitlements (Figure 3c). The gender gap persists when we control for other nonneed factors (Table 4, Column 5). In order to better understand how gender plays, we rerun the regressions from Analyses A, B, and to C including not only gender and a dummy for living with one's partner and also the interaction between the two. ^17^Results are reported in Table B.III in Appendix B, Data S1. When singles, women use more formal care than men for given entitlements. As (single) elderly women may be more likely to receive support from their network than elderly men (see, e.g., Andrew, 2005), their higher care use may be in line with the idea that informal support may be a catalyst for formal care use (cf. Schmidt, 2017).

However, when they have a partner, women have lower entitlements and use less home care than their male counterparts, for given needs (negative interaction terms in Columns 2A and 2B of Table B.III in Appendix B, Data S1). For men instead, having a partner is not found to be an entitlement‐reducing factor (Column 2B). These patterns are at odds with earlier studies, which report that (in different contexts) women with a partner are more likely to receive professional home care than men with a partner, as the latter resort more to spousal informal care than the former (Arber & Ginn, 1991; Schmidt, 2017). We are unable to uncover whether, in the Netherlands, women are less likely to apply for home care when they have a partner at home, or whether there is some unequal treatment of nonsingle applicants, depending on their gender.

Turning to geographical disparities, the largest difference in need‐standardized use between two of the 10 CIZ offices is €560 (compared with an average use of €683), as shown in Figure B.2 in Appendix B Data S1. There are also sizeable differences between CIZ regional offices in need‐standardized entitlements, which range from €1,104 to €2,023. These differences are also visible in the point estimates for use (Table 4), meaning that they do not merely reflect composition effects. Under the assumption that no major need factor is left unobserved, this pattern is suggestive of substantial variation in the eligibility process across CIZ regional offices in spite of the centralization of the agency. Furthermore, differences across CIZ offices at the stage of the conversion of entitlements into use are also observed and do not disappear when we control for socioeconomic and demographic factors and for LTC purchasing region. As CIZ regional offices do not correspond to any political or administrative entity that could influence the supply of care, the disparities across offices might well pick up local variation in local norms or preferences regarding elderly care. ^18^Comparing need‐standardized use and entitlements across the 32 LTC purchasing regions (Figure B.3 in Appendix B, Data S1) also points to sizeable variation. Although statistical precision is low, this result echoes the findings from Duell et al. (2019) for nursing home care use. Finally, need‐standardized use varies with the size of the municipality of residence (Figure 3), but such variation is explained by systematic differences in the populations of these areas use (Table 4). We still observe entitlements to be €191 lower for elderly in rural areas, even though applications for a need assessment are made online or by mail—meaning that the elderly living further away from CIZ regional offices do not incur higher application costs.

## 6 ROBUSTNESS CHECKS AND DISCUSSION

### 6.1 Robustness checks

For Analyses A and B, the estimation of horizontal inequity relies on a statistical derivation of needs. We perform two main checks to gauge the robustness of our baseline estimates.

First, we take an alternative normative stance about intrahousehold informal caregiving: We no longer consider it a legitimate substitute for formal home care. When replicating the analysis considering household size, having a coresiding partner as well as the interaction between gender and having a partner as nonneed factors rather than need variables point estimates for HI become more negative (Table 5, “Check 1”). According to the baseline regression estimates of Analysis B, for given needs and nonneed factors, elderly with a partner receive €385 (25%) lower entitlements than single living elderly (Table B.I, Column 3, in Appendix B, Data S1). These estimates suggest that the lower use of home care by the elderly with a partner alive in the Netherlands is partly due to their lower home care entitlements, as hypothesized by Bakx, De Meijer, Schut, and Van Doorslaer (2015). If this difference between singles and nonsingles is reclassified as inequitable, pro‐poor horizontal inequity at the stage of LTC eligibility will be larger, as single‐living elderly, and in particular single women, have a lower income. ^19^On the other hand, the elderly living in larger households have higher disposable income while receiving lower entitlements and care (Table B.I). The pro‐rich effect of including household size as a need variable does not however offset the pro‐poor effect of taking the presence of the spouse also as a need variable. Similarly, for Analysis A, the lower care use of the elderly with a partner explains why considering household composition as need variables attenuates pro‐poor inequity.

**TABLE 5:** CI and HI for Analyses A and B—robustness checks

	Variant	CI (outcome)	HI (outcome)
Analysis		(1)	(2)
A. Home care use (equity overall)	Baseline	−0.340^***^ [−0.361, −0.315]	−0.036^***^ [−0.057, −0.010]
	Check 1: having a partner is a nonneed factor		−0.058^***^ [−0.066, −0.017]
	Check 2: no administrative data on health care use		−0.043^***^ [−0.077, −0.035]
B. Home care entitlements (equity at eligibility stage)	Baseline	−0.288^***^ [−0.311, −0.267]	−0.016^n.s.^ [−0.036, −0.005]
	Check 1: having a partner is a nonneed factor		−0.025^*^ [−0.041, −0.000]
	Check 2: no administrative data on health care use		−0.020^**^ [−0.045, −0.005]

*Note*: Asymmetric 95% confidence intervals in brackets, computed using a cluster‐bootstrap approach (1,000 replications; see Data S3).

Abbreviations: CI, confidence interval; HI, horizontal inequity.

^***^ *p* < 0.01.

^**^ *p* < 0.05.

^*^ *p* < 0.10.

^n.s.^ *p* ≥ 0.10.

Second, we replicate Analyses A and B excluding the dummies for pharmaceuticals use and the costs incurred for different types of health care from the list of need factors before running the Lasso selection procedure. Indicators of health care use might indeed already reflect some inequalities in health care use, which may be correlated with inequalities in LTC use, and thus filter out some of the differences that we aim to explain. The estimates (“Check 2”) are similar to the baseline analysis and not statistically different from them.

Four additional robustness checks are provided in Table E.I in Appendix E, Data S1. Check 3 (in which the Lasso procedure is used to select both need and nonneed variables), Check 4 (in which unobserved factors correlating with income are assumed to be need, rather than nonneed, factors), and Check 5 (in which household income is equivalized using a different scale) deliver estimates of HI very close to baseline results. In a last variant, we relax the assumption that income is shared equally within the household. On the basis of estimates from Cherchye, De Rock, and Vermeulen (2012), we allocate 63% of the couple's income to women. Entitlements remain equitably allocated along the income distribution, but the gradient in the conversion of entitlements into use becomes slightly pro‐rich. This is because women, who were found to convert more of their entitlements into use than men on average, are reranked higher in the distribution of income. We take the results from this last scenario with caution, as its underlying assumptions may not be valid in our context. ^20^See Appendix E, Data S1 for more details. Still, it provides a relevant illustration of how a revision of income ranking (here, of men vs. women and widows vs. couples) may affect our estimates of socioeconomic inequity in care access. More generally, one additional interest of the estimates of inequity along dimensions other than income (cf. Section 5.3) is that they allow to gauge how SES inequity would shift if the economic resources of some groups were to be revised.

### 6.2 Discussion of limitations and interpretation

Our study has two data‐related limitations. First, we are not able to look separately at the probability of requesting eligibility and at the need assessment itself: We cannot rule out that income‐related differences in the propensity to request an assessment are offset by differential treatment of applicants through the need assessment.

Second, we can only study home care use: We are unable to unveil potential inequity in nursing home use because the survey does not include the institutionalized population. In the Netherlands, home care users represent about two third of the 65 and older population receiving LTC, but nursing home care still accounts for 70% of LTC spending (Muir, 2017). Moreover, the focus on home care might induce a selection bias if nonneed factors influence eligibility for institutional care after need factors are controlled for. To shed light on whether this may be the case, we enrich our dataset with register data on eligibility for institutional care in 2013. We find evidence that a low income, having children (nearby), and education correlate with the probability to have become eligible for institutional care in January 2013, conditional on needs. ^21^Recall that the survey was conducted in the Fall 2012. If lower income elderly are more likely to be selected out of our sample, the pro‐poor gradient in home care use, conditional on needs, may be underestimated.

However, differential probability to be eligible for nursing home will result in a selection bias only if the unobserved determinants of (non)eligibility for institutional care correlate with unobserved determinants of home care use. Our analysis controls for a very rich set of variables relating to health and functional status, arguably limiting the scope for unobserved health determinants of eligibility and use. Given the way the needs assessment is organized in the Netherlands, we also expect preferences for some type of care not to play a role in the decision to make an applicant eligible for home care rather than for institutional care.

Another form of sample selection may be induced by differential mortality across population groups, which could theoretically bias our equity estimates. In particular, low‐income individuals and men have a lower life expectancy, on average. The economic literature has come up with methods to correct income inequality estimates for “the missing poor” problem (Lefebvre, Pestieau, & Ponthière, 2013). In the case of equity in care use, however, we are not aware of any method allowing to adjust for differential mortality. Even speculating about the direction of the bias it might induce would require making a series of (heroic) assumptions regarding the relationship between care needs and mortality and how such relationship might differ across income, gender, and so forth.

Our analysis focuses on publicly subsidized home care. It includes informal care, in two indirect ways. First, by considering the partial correlation between household composition and entitlements as a legitimate source of inequality in home care access, our baseline analysis incorporates the usual care expected from household members. Second, LTC vouchers in the Netherlands can be used to hire either noncontracted providers or relatives. As such, compensated informal care is considered a legitimate substitute for formal care and is included in our measure of home care use. Inclusion is implicitly based on the assumption that compensated informal care has a value as high as that of professional home care. If it were not the case (e.g., because informal caregivers might be unskilled), the value of home care received by voucher recipients would be overestimated. How this would affect our (socioeconomic) inequity estimates depends on which (income) groups are more likely to use vouchers to compensate relatives in the Netherlands, on which we have no insight. ^22^We have checked that our main result—of a negative gradient in home care use, conditional on needs, and entitlements—is robust to assuming that all vouchers are used to compensate informal with imputed value set to 0. This is because vouchers represent a limited share of publicly financed home care use (11% in the bottom income decile and 20% in the top decile).


Our study does not include uncompensated informal care receipt, for which we have no data. The normative premise underlying the Dutch LTC system in 2012 was that access to publicly subsidized care should be independent from the provision of uncompensated informal care beyond “usual care” availability. As such, ignoring uncompensated informal care does not come as a limitation, given our objective to assess equity in access to home care in the context of the social insurance scheme. Documenting the joint receipt of informal care and formal care would however contribute to understanding disparities in home care use by showing which subpopulations substitute formal care with voluntary informal care.

## 7 CONCLUSION

When public insurance coverage is limited, LTC use risks being very pro‐rich distributed. The Netherlands has tried to overcome this risk by making such coverage compulsory and comprehensive and by formalizing the needs‐based allocation of care. We examine the extent to which this attempt has proven successful. Combining survey information and administrative register data, we test for horizontal inequity in home care access in the Netherlands. In addition, we disentangle inequalities in two stages: when home care eligibility decisions are made and when care entitlements are converted into actual use.

We find clear evidence of a pro‐poor gradient in home care use after controlling for care needs. The main reason for this pattern is that poorer elderly convert a larger share of their entitlements into actual use, consistent with Tenand et al. (2020). Assuming no supply constraints, the negative income gradient in home care use would likely be caused by differences in individuals' choices. One such explanation might be a nonzero price elasticity of home care demand (Non, 2017; Roquebert & Tenand, 2017), as the copayment schedule translates into a marginal price of home care higher for richer beneficiaries. However, available evidence does not allow to conclude whether the lower use of home care by higher income elderly results from user behavior or from the functioning of the LTC system.

The critical finding of our study is that the need assessment dampens inequity in access by SES. We do not find any evidence that higher income, wealthier, or higher educated elderly are able to better navigate the needs assessments procedure. Those elderly are not more likely to claim—or to be granted—home care entitlements at an earlier stage of the disablement process.

Still, we find some inequity in entitlements along other dimensions, as some population groups receive higher or lower home care entitlements given needs. Elderly with a non‐Western migrant background seem to receive more home care entitlements but use less of them. Finally, quite large differences across CIZ regional offices in the entitlements for given needs and in the conversion of these entitlements into use are observed, despite the Dutch need assessment agency being theoretically centralized. We are unable to unravel whether this reflects local differences in preferences or in the provision of informal care, systematic variation in the supply of home care across municipalities, or differences in practices across CIZ offices.

The relevance of our study extends beyond home care in the Netherlands: Horizontal equity in access to virtually all LTC services and medical care depends on (1) whether population groups have equal access to diagnosis and “treatment” decisions and (2) whether they receive the same treatment for a given diagnosis. Usually, these two cannot be separated in empirical studies, but the Dutch context and data make it an ideal case to identify the stage(s) at which inequity occurs.

Furthermore, the Dutch example is of specific interest for other countries aiming to distribute LTC according to needs such as Germany, France, Spain, and also South Korea and Japan (OECD, 2020). These countries vary in both “the *criteria* under which a given individual is deemed eligible for publicly funded support for long‐term care” and “the process involved in selecting from the general population those who receive this support and determining for how much support each person is eligible” (Eleftheriades & Wittenberg, 2013). There is little empirical evidence available on what types of procedures do favor equity in access. On the basis of our results from the Netherlands, we speculate that entrusting the needs assessment to an independent agency, in which assessors rely on extensive guidelines and do not collect income or wealth information, is effective in limiting socioeconomic inequity in LTC access.

Distinguishing between equity in eligibility and equity in entitlements however shows that setting equitable eligibility rules for LTC is not a sufficient condition for equitable use, as uptake of benefits is not guaranteed. The Dutch case illustrates that, even in a comprehensive public system in which financial barriers to access are limited, the distribution of LTC use may deviate from the distribution of needs. A better understanding of the sources of inequalities helps in identifying any need for corrective policies and their appropriate design.

Although our analysis suggests that the Dutch LTC system was fairly successful in allocating home care services independently from the ability to pay for such services in 2012, the situation may have changed since the system was profoundly reformed in 2015. Home care is now funded under different schemes: Eligibility for and financing of community‐based nursing care are the responsibilities of regional health insurers, whereas other home care services are entrusted to municipalities, which can set their own eligibility criteria. This setting may not only increase geographical and socioeconomic inequity in home care entitlements but also implies that administrative data on eligibility decisions are no longer available, making it impossible to replicate our analysis for the postreform period. Additionally, whether the recent removal of (income‐dependent) copayments on home care has fostered a higher use among richer beneficiaries is a question for future research. Despite all these reforms, equity in LTC use remains an important policy goal, and a better understanding of how decisions on the use of LTC come about remains crucial in achieving it.

## FUNDING

Access to the data was in part funded by the Open Data Infrastructure for Social Science and Economic Innovations (ODISSEI). The authors acknowledge additional financial support from the Network for Studies on Pensions, Aging and Retirement (Netspar) within the project “Optimal saving and insurance for old age: the role of public long‐term care insurance”.

## ACCESS TO AND USE OF INDIVIDUAL‐LEVEL DATA

The results presented in this article are based on calculations by the authors using non‐public microdata from Statistics Netherlands (CBS). The datasets used include the *Gezondheidsmonitor Volwassenen en Ouderen* 2012, provided by the GGDs, CBS and RIVM. Under certain conditions and a confidentiality agreement, these microdata are accessible for statistical and scientific research. For further information: microdata@cbs.nl. Exploitation of the data and publication of the results are made in compliance with the European privacy legislation (GDPR, May 25^th^, 2018).

## CONFLICT OF INTERESTS

The authors have no conflict of interests to declare.

## Supporting information

Data S1: Supplementary materials
