# Supplementary material for: Eligibility or use? Disentangling the sources of horizontal inequity in home care receipt in the Netherlands
Source: Health Econ. 2020 Jul 8;29(10):1161–79. doi: 10.1002/hec.4126 (PMC7540300; doi:10.1002/hec.4126)
Supplement: Supplementary file 1 — Data S1: Supplementary materials [file HEC-29-1161-s001.pdf]

Supplementary material

Eligibility or use? Disentangling the sources of horizontal inequity in home care receipt in the Netherlands

Marianne Tenand[1], Pieter Bakx[2] and Eddy van Doorslaer[3]

April 2020

<u>Supplementary material to the article:</u>

Tenand, M., Bakx, P. & van Doorslaer, E. (2020). “Eligibility or use? Disentangling the sources of horizontal inequity in home care receipt in t Netherlands”, ***Health Economics.*** DOI: 10.1002/hec.4126

[1] Erasmus School of Health Policy & Management (ESHPM), Erasmus University Rotterdam, the Netherlands. Corresponding author. Contact: tenand[at]eshpm.eur.nl or marianne.tenand.pro[at]gmail.com.
[2] Erasmus School of Health Policy & Management (ESHPM), Erasmus University Rotterdam, the Netherlands.
[3] Erasmus School of Health Policy & Management (ESHPM) and Erasmus School of Economics (ESE), Erasmus University Rotterdam, the Netherlands.

Table of contents

Appendix A: implementation of the Lasso procedure p. 3

A.1. Selection of need variables and of and non-need factors prior to the baseline estimation of HI for Analyses A and B p. 3

A.2. Selection of need variables and of and non-need factors prior to the baseline estimation of HI for Analyses A and B p. 4

Appendix B: Additional results p. 8

Appendix C: Inference on concentration and horizontal inequity indexes p. 15

C.1. Standard error of the concentration index of LTC use and LTC entitlements p. 15

C.2. Standard error of the concentration of needs, the horizontal inequity index and the contributions of non-need factors p. 16

Appendix D: Deriving and decomposing the horizontal inequity index p. 19

D.1. Decomposition of HI^A and HI^B p. 19

D.2. Decomposition of HI^C p. 20

Appendix E: Additional robustness checks p. 22

Appendix F: List of datasets used p. 26

Additional References p. 28

Appendix A: implementation of the Lasso procedure

A.1. Selection of need variables and of and non-need factors prior to the baseline estimation of HI for Analyses A and B

The computation of the horizontal inequity indexes in Analyses A and B relies on linear predictions from regression analyses (Equations (1.A) and (1.B)). The statistical precision of the estimates of HI will in turn depend on the statistical precision of the regression estimates. Our dataset contains a very large number of (potentially highly correlated) need variables that may be included as regressors in Equations (1.A) and (1.B). In order to maximize statistical efficiency, we implement a data-driven method to select a subset of need variables to be included in the regression analyses.

We implement a Lasso procedure similar to the one performed by Bakx *et al.* (2018),[4] following the methodology proposed by Tibshirani (1996). The Lasso aims at selecting covariates in a regression model so that the model achieves a balance between low bias and high statistical efficiency. The Lasso consists in a procedure of shrinkage, whereby coefficients of covariates that are not, or weakly, associated with the outcome variable are shrunk towards zero and eliminated from the regression analysis. It relies on the minimization of an objective function that penalizes the absolute size of the regression coefficients. As the penalizing factor increases, the coefficients of all covariates shrink further towards zero; as it decreases, the procedure is more lenient towards covariates with a small partial correlation with the outcome.[5]

We apply the Lasso procedure separately to the following variants of Equations (1.A) and (1.B):

$$U_i = \tilde{\alpha}_0 + \tilde{X}_i' \tilde{\alpha}^X + \tilde{\varepsilon}_i \qquad (A.A.1)$$

$$E_i = \tilde{\beta}_0 + \tilde{X}_i' \tilde{\beta}^X + \tilde{u}_i \qquad (A.B.1)$$

[4] A more detailed description of the procedure can be found in Appendix C of Bakx *et al.* (2018).
[5] See Bakx *et al.* (2018) for additional details. We follow their choice of using the objective function and penalization proposed in Belloni *et al.* (2012). For all 3 analyses we have performed the Lasso selection without taking into account the survey structure. To the best of our knowledge, no specific adjustment of the standard Lasso procedure to complex sampling design survey data has been well established so far.

where $\tilde{X}_i$ is a vector containing the full list of the need variables prior to performing the Lasso selection, for individual i.

The outcome of the Lasso procedures that we apply to Equations (A.A.1) and (A.B.1) is made of two sub-lists of need variables (two sub-vectors of $\tilde{X}$). As there is no theoretical ground for the legitimate needs for home care entitlements and the needs for home care use to be different, we define our final list of need variables as the *union* of the variables selected by the Lasso procedures in Analysis A and Analysis B.

Table A.I provides the list of the variables that were included as need factors in the Lasso procedures. It also indicates which of these variables were selected and included in the regression analyses implemented to derive the horizontal inequity indexes.

A.2. Selection of need variables and of and non-need factors prior to the baseline estimation of HI for Analyses A and B

In a robustness check, we use a Lasso to select both a subset of need variables and a subset of non-need factors. We apply the Lasso procedure separately to the following variants of Equations (1.A) and (1.B):

$$U_i = \tilde{\alpha}_0 + \tilde{X}_i'\tilde{\alpha}^X + Z_i'\alpha^Z + \tilde{\varepsilon}_i \qquad (A.A.3)$$

$$E_i = \tilde{\beta}_0 + \tilde{X}_i'\tilde{\beta}^X + Z_i'\beta^Z + \tilde{u}_i \qquad (A.B.4)$$

In this robustness check, the outcome of the Lasso procedures applied to Equations (A.A.1) and (A.B.1) is made of two times two sub-lists (two sub-vectors of $\tilde{X}$ and two sub-vectors of Z). We define our final list of need variables as the *union* of the variables selected by the Lasso procedures in Analysis A and Analysis B.[6]

[6] The list of variables selected is available on request.

Table A.I: Need variables in the Lasso procedure (1/2)

Variables used as inputs in the Lasso procedure:	Variables selected by the Lasso procedure: *(A) LTC use*	*(B) LTC entitlements*
	(1)	(2)
Socio-demographic characteristics		
Age (5-year categories)	Yes	Yes
Having a partner in the household	Yes	Yes
Number of additional household members	Yes	No
Functional limitations[a,c]		
Follow a conversation with 3 persons or more	Yes	Yes
Engage into a conversation with 1 person	Yes	Yes
Read the small characters in the newspaper	No	Yes
Recognize someone from a 4 meter distance	Yes	Yes
Carry an object of 5 kilos over 10 meters	Yes	Yes
Bend to reach for something on the ground	Yes	Yes
Walk 400 meters without resting	Yes	Yes
Hearing problems	Yes	Yes
Sight problems	Yes	Yes
Mobility problems	No	No
Self-reported health and mental health[,c]		
Self-reported health: bad, average or good	Yes	Yes
Loneliness (no, moderately, severely, very severely)	No	No
Feeling depressed	No	No
Anxiety or depression (low *versus* moderate to high)	No	Yes
Chronic condition[b,c]		
Chronic disease: diabetes	No	No
Chronic disease: cardiovascular (currently or ever)	No	No
Chronic disease: heart failure or other heart disease (currently or ever)	No	No
Chronic disease: cancer (currently or ever)	No	No
Chronic disease: high blood pressure or small vessels	Yes	Yes
Chronic disease: COPD	No	No
Chronic disease: bowel disorder or incontinence	Yes	Yes
Chronic disease: arthrosis or arthritis	Yes	Yes
Chronic disease: hernia or back issues	Yes	Yes
Chronic disease: neck, shoulder, wrist or hand issues	No	No
Chronic disease: eczema or psoriasis	No	No
Chronic disease: migraine	No	No
Chronic disease: dizziness	Yes	Yes
Chronic disease: other	No	Yes

Notes: [a] for each activity, the respondent could answer: no difficulty, some difficulties, major difficulties, cannot do.
[b] for each condition, the respondent could answer: yes or no.
[c] missing values were coded as an additional category for each of the functional limitations and self-reported chronic diseases.

Table A.I: Need variables in the Lasso procedure (2/2)

Variables used as inputs in the Lasso procedure:	Variables selected by the Lasso procedure: (A) LTC use	(B) LTC entitlements
	(1)	(2)
Health care costs incurred in 2011		
GP costs	Yes	Yes
Pharmacy costs	Yes	Yes
Paramedical costs	Yes	Yes
Dental care costs	No	No
Hospital care costs	No	No
Health care costs incurred abroad	No	No
Costs for first-line psychological care	No	No
Mental health care costs	No	No
Geriatric care costs	No	No
Costs on assistive devices (*hulpmiddel*)	Yes	Yes
Other health care costs	No	No
Total health care costs	No	Yes
Drug use in 2011[d]		
ATC A06 (Drugs for constipation)	Yes	Yes
ATC A12 (Mineral supplements)	No	Yes
ATC B01 (Antithrombotic agents)	Yes	Yes
ATC B03 (Anti-anemic preparations)	No	Yes
ATC C03 (Diuretics)	Yes	Yes
ATC D02 (Emollients and protectives)	Yes	Yes
ATC J06 (Immune sera and immoglobulins)	Yes	Yes
ATC M01 (Anti-inflammatory and anti-rheumatic products)	Yes	Yes
ATC N05 (Psycholeptics)	Yes	Yes
ATC N06 (Psychoanaleptics)	Yes	Yes
ATC R01 (Nasal preparations)	Yes	Yes
ATC Y (ATC code not filled in)	Yes	Yes

Notes: [d] There exists additional ATC codes that are not reported, as they were never selected by the Lasso procedure. The full list of ATC provided in Bakx *et al.* (2018).

Table A.II: Non-need factors in the regression analysis (Analyses A, B and C)

Socio-economic status	
Education	(1) primary education
	(2) secondary education
	(3) higher education
	(4) unknown
Disposable income	10 deciles
Per capita wealth	10 deciles
Homeownership	(1) no
	(2) yes
Demographic characteristics	
Gender	(1) woman
	(2) man
Origin (*born in a foreign country or*	(1) Netherlands
having a parent born in a foreign	(2) other Western country or Dutch Indies
country)	(3) other non-Western country
Place of residence	
Size of the municipality of residence	(1) Less than 10,000 inhabitants
	(2) between 10,001 and 50,000 inhabitants
	(3) between 50,001 and 150,000 inhabitants
	(4) more than 150,000 inhabitants
CIZ region	1 of the 10 administrative regions defined for needs assessments
LTC purchasing region	1 of the 8 groups of the 32 administrative regions in charge of the provision of LTC[a]
Family characteristics	
Number of children	Continuous variables
Number of daughters	Continuous variables
Closest child	(1) no child
	(2) co-resides
	(3) lives in the same municipality
	(4) lives in a different municipality

Notes: [a] see Online Appendix C, section C.2 for explanations regarding the grouping of the regions.

Appendix B: Additional results

Table B.I: Regression estimates for Analyses A and B (1/2)

	Analysis A		Analysis B	
Outcome:	*LTC use (value)*		*LTC entitlements (value)*	
	Coef.	Std. error	Coef.	Std. error
	**(1)**	**(2)**	**(3)**	**(4)**
Need variables				
Age: 65-69	-129.9*	77.0	-397.3**	154.9
Age: 70-74	-268.7***	76.1	-579.1***	146.2
Age: 75-79	-236.5***	76.1	-436.1***	125.6
Age: 80-84	*Ref.*	-	*Ref.*	-
Age: 85-89	827.6***	178.0	1410.6***	241.8
Age: 90-94	2384.7***	419.1	3822.1***	700.1
Age: 95+	5773.9***	1092.8	7495.0***	1483.1
Partner in the household	-213.1***	62.5	-384.9	236.0
Number of additional household members	-99.1*	52.5	20.8	213.8
Follow a conversation: no difficulty	28.3***	55.2	-30.0	98.5
Follow a conversation: slight to moderate difficulty	*Ref.*	-	*Ref.*	-
Follow a conversation: serious difficulty	1239.5***	347.5	1901.2***	462.7
Engage into a conversation: no difficulty	-303.8***	110.2	-536.0***	179.9
Engage into a conversation: moderate difficulty	2414.8***	889.3	3888.9***	1476.4
Engage into a conversation: slight or serious difficulty	*Ref.*	-	*Ref.*	-
Recognize someone from a distance: no difficulty	-213.4	173.1	-236.6	249.5
Recognize someone from a distance: slight to serious difficulty	*Ref.*	-	*Ref.*	-
Read the small characters: no to moderate difficulty	*Ref.*	-	*Ref.*	-
Read the small characters: serious difficulty	735.7***	217.7	1081.6	414.2
Carry an object of 5 kg: no or moderate difficulty	*Ref.*	-	*Ref.*	-
Carry an object of 5 kg: slight difficulty	-306.6***	96.3	-341.8***	127.1
Carry an object of 5 kg: serious difficulty	894.6***	172.6	1593.1***	278.5
Bend to reach the ground: no difficulty	-380.9**	152.2	-617.0**	295.8
Bend to reach the ground: slight difficulty	-371.7**	172.8	-555.3*	313.1
Bend to reach the ground: moderate difficulty	*Ref.*	-	*Ref.*	-
Bend to reach the ground: serious difficulty	2423.0***	458.5	3956.0***	740.1
Walk 400 meters: no difficulty	112.9	89.9	-91.0	131.2
Walk 400 meters: slight to moderate difficulty	*Ref.*	-	*Ref.*	-
Walk 400 meters: serious difficulty	704.8***	217.5	1155.7***	288.5
Hearing limitations: no or unknown	*Ref.*	-	*Ref.*	-
Hearing limitations: yes	233.0	236.1	457.1	420.9
Sight limitations: no	-37.9	237.1	20.3	305.0
Sight limitations: yes or unknown	*Ref.*	-	*Ref.*	-
Self-reported health: v. good/good/average	*Ref.*	-	*Ref.*	-
Self-reported health: v. bad/bad	957.1***	181.6	1637.0***	264.1
Anxiety or depression: low chance	69.9	38.0	17.0	81.0
Anxiety or depression: low chance or unknown	*Ref.*	-	*Ref.*	-

Continued on next page

Table B.I: Regression estimates for Analyses A and B (2/2)

	Analysis A		Analysis B	
Outcome:	*LTC use (value)*		*LTC entitlements (value)*	
	Coef.	Std. error	Coef.	Std. error
	**(1)**	**(2)**	**(3)**	**(4)**
Continued from previous page				
Blood disease: no	326.7***	60.6	507.9***	72.3
Bowel disorders or incontinence: no	-266.3***	59.3	-552.5***	106.3
Arthritis or arthrosis: no	242.8***	61.7	449.2***	95.6
Hernia: no	100.6	109.4	148.7	198.4
Hernia: yes	-358.4***	102.7	-729.0***	191.6
Hernia: unknown	*Ref.*	-	*Ref.*	-
Dizziness: yes	371.2***	131.3	662.5***	227.0
Chronic disease: other (miscellanea)[a]	42.0	90.1	220.6	126.0
GP costs[b]	3911.3***	1100.9	5859.0***	1068.5
Pharmacy costs[b]	82.2***	21.4	101.0**	30.8
Paramedical costs[b]	1046.3***	254.9	1408.0***	237.6
Costs on medical devices[b]	799.4***	155.1	991.6***	129.2
Total health care costs[b]	-12.3	7.8	3.2	16.9
ATC A06 (Constipation treatments)	224.8***	76.3	359.9***	108.8
ATC A12 (Mineral supplements)	115.6	152.2	310.4	227.2
ATC B01 (Antithrombotic agents)	-2.2	58.4	92.3	92.6
ATC B03 (Anti-anemic preparations)	67.9	182.6	420.8*	253.5
ATC C03 (Diuretics)	93.9	50.2	63.1	90.5
ATC D02 (Emollients and protectives)	416.4***	116.2	470.2***	157.8
ATC J06 (Immune sera and immoglobulins)	-2646.2***	513.7	-3661.2***	954.9
ATC M01 (Anti-inflammatory and anti-rheumatic products)	-294.6***	86.1	-433.4***	116.6
ATC N05 (Psycholeptics)	371.4	259.1	1327.5***	385.0
ATC N06 (Psychoanaleptics)	674.6***	98.0	1338.2***	170.9
ATC R01 (Nasal preparations)	-341.8***	90.6	-527.8***	116.7
ATC Y (ATC code not filled in)	1855.7**	778.6	5286.1***	1594.4
Non-need factors *(see Table IV and B.II)*	Yes		Yes	
Constant	-281.8	260.4	-23.7	560.9
**N**	154,646		154,646	
R^2	0.158		0.166	

Notes: *** *p < 0.01*, ** *p < 0.05*, * *p < 0.10*, n.s. *p ≥ 0.10*. [a]The full list of need variables and their coding before the Lasso selection is displayed in Table A.I (Online Appendix A). [b]Health care costs are expressed in thousands euros. Outcomes are expressed in euros over year 2012. Weighted linear estimations of Equations (1.A) for Columns (1) and (2) and (1.B) for Columns (3) and (4) (defined in Section 3). The estimations take into account the clustered design of the sample (335 primary sampling units). When performing the estimations, a few observations for which the primary sampling unit is missing have to be dropped.

Table B.II: Regression estimates for long-term care regions - Analyses A, B and C

	**Analysis A**		**Analysis B**		**Analysis C**	
Outcome:	*LTC use (value)*		*LTC entitlements (value)*		*LTC use (value)*	
	Coef.	Std-error	Coef.	Std-error	Coef.	Std-error
	(1)	(2)	(3)	(4)	(5)	(6)
CIZ regional office 1	*Ref.*	-	*Ref.*	-	*Ref.*	-
CIZ regional office 2	179.1^{***}	53.1	34.5	90.1	1854.0^{***}	440.2
CIZ regional office 3	51.1	51.5	-237.0^{**}	129.1	1864.4^{**}	784.0
CIZ regional office 4	-32.7	62.3	-282.8^{**}	144.1	1110.0^{*}	579.7
CIZ regional office 5	-0.4	51.3	-228.5^{**}	99.1	1129.2^{**}	480.2
CIZ regional office 6	-71.1	57.9	-311.2^{***}	117.2	876.8^{*}	490.5
CIZ regional office 7	458.9^{***}	150.4	424.4^{*}	217.2	1228.8^{*}	668.7
CIZ regional office 8	816.9^{***}	262.9	756.0^{**}	330.8	2490.8^{**}	999.4
CIZ regional office 9	310.3^{**}	122.9	359.2^{**}	159.2	1222.7	751.9
CIZ regional office 10	-74.2	68.6	-218.9	133.6	762.6	518.1
Group of purchasing regions 1 (lowest use)	*Ref.*	-	*Ref.*	-	*Ref.*	-
Group of purchasing regions 2	127.2	169.4	-19.5	257.3	-372.8	699.6
Group of purchasing regions 3	198.1	171.1	60.9	262.2	-306.5	776.0
Group of purchasing regions 4	321.3^{*}	165.6	271.9	257.8	251.9	784.9
Group of purchasing regions 5	401.8^{**}	164.2	414.9	258.3	397.7	766.8
Group of purchasing regions 6	475.0^{***}	162.1	587.0^{**}	226.1	440.0	697.0
Group of purchasing regions 7	603.5^{***}	184.3	719.8^{***}	264.6	1049.8	727.1
Group of purchasing regions 8 (highest use)	729.3^{***}	176.8	912.4^{***}	261.0	1153.2	800.0
Other non-need factors *(see Table IV)*	Yes		Yes		Yes	
Need factors *(see Table B.I)*	Yes		Yes		No	
Home care entitlements *(see Table IV)*	No		No		Yes	
**N**	154,646		154,646		14,136	

Notes: *** $p < 0.01$, ** $p < 0.05$, $^{*}p < 0.10$, $^{n.s.}$ $p \geq 0.10$. Outcomes are expressed in euros over year 2012. Weighted linear estimations of Equations (1.A) for Columns (1) and (2) and (1.B) for Columns (3) and (4) (defined in Section 3). The estimations take into account the clustered design of the sample (335 primary sampling units). When performing the estimations, a few observations for which the primary sampling unit is missing have to be dropped.

Figure B.1: Average standardized outcomes, by wealth decile (Analyses A, B and C)

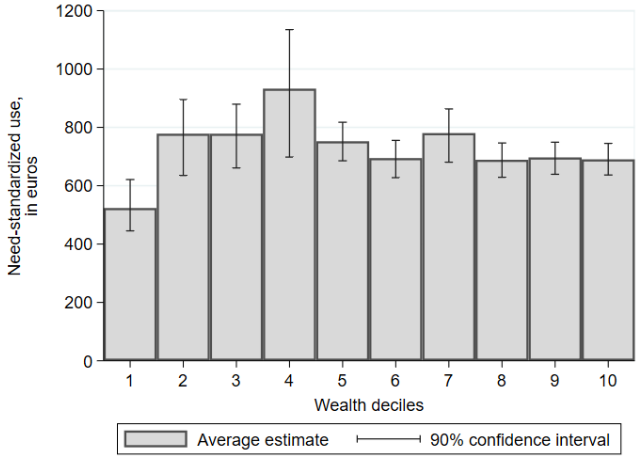

Panel A: Average need-standardized use by wealth decile (Analysis A)

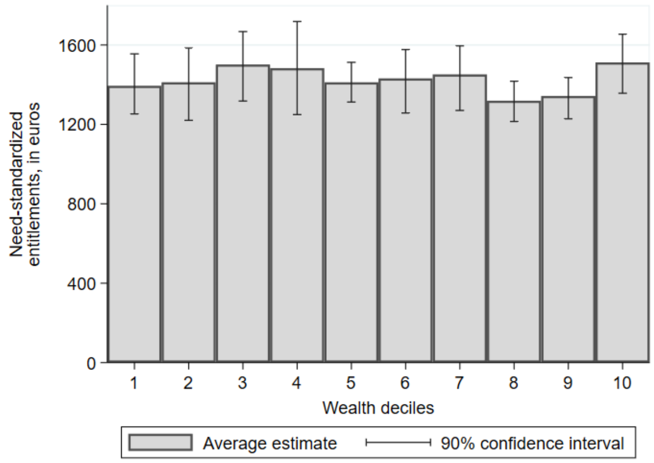

Panel B: Average need-standardized entitlements by wealth decile (Analysis B)

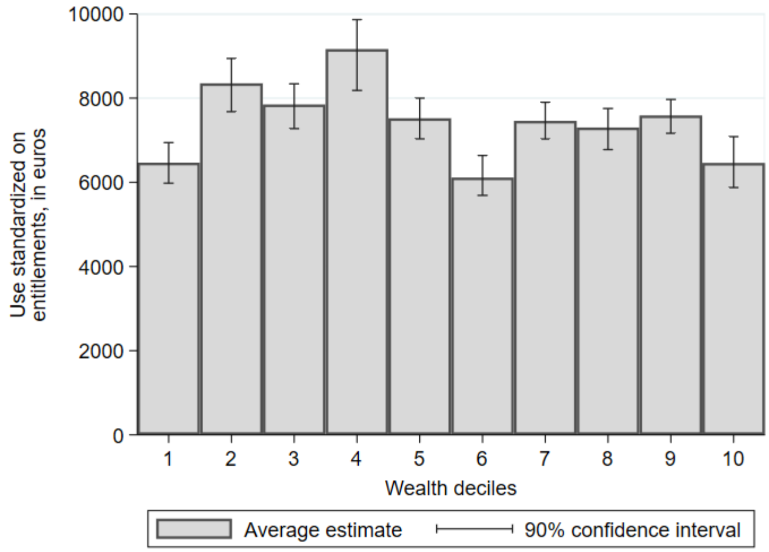

Panel C: Average use standardized for entitlements by wealth decile (Analysis C)

Notes: Need-standardized use and entitlements (Analyses A and B) and use standardized for entitlements (Analysis C) are defined in Equations (3.A) to (3.B). Asymmetric 90%-level confidence interval based on 1,000 Bootstrap replications (see Online Appendix C). Values expressed in euros over 2012.

Figure B.2: Average standardized outcomes, by CIZ regional office (Analyses A, B and C)

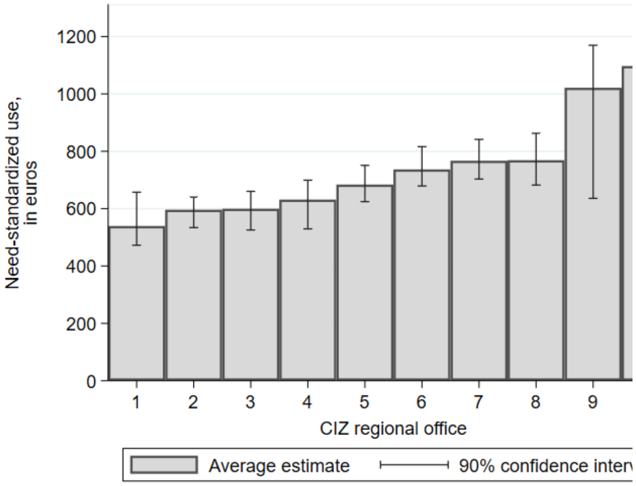

Panel A: Average need-standardized use by CIZ regional office (Analysis A)

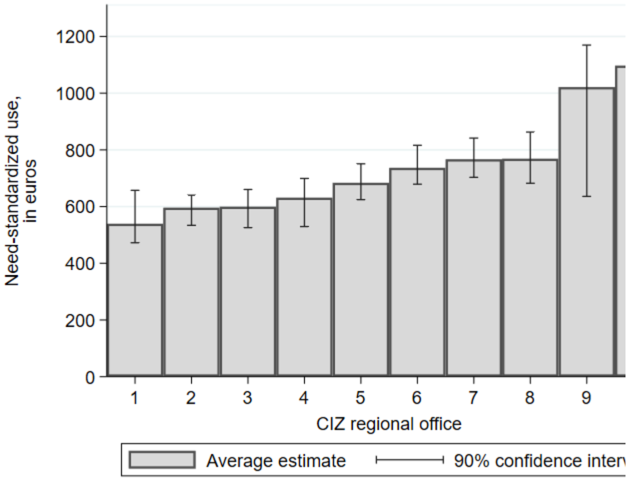

Panel B: Average need-standardized entitlements by CIZ regional office (Analysis B)

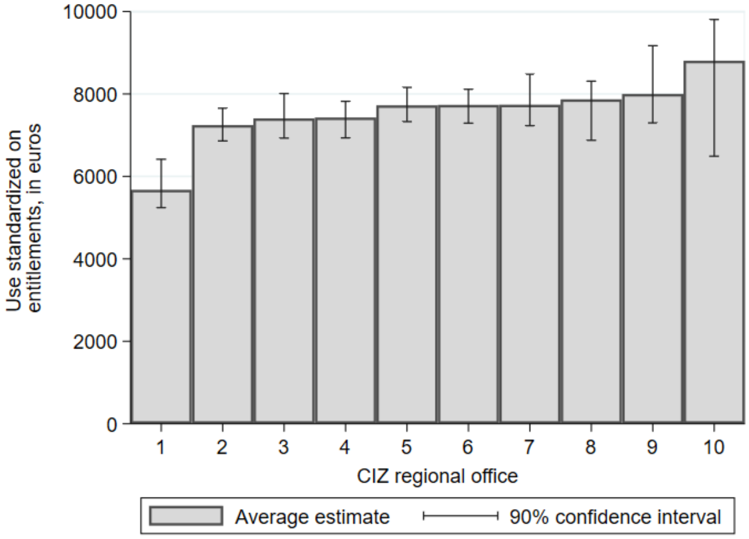

Panel C: Average use standardized for entitlements by CIZ regional office (Analysis C)

Notes: Need-standardized use and entitlements (Analyses A and B) and use standardized for entitlements (Analysis C) are defined in Equations (3.A) to (3.B). Asymmetric 90%-level confidence interval based on 1,000 Bootstrap replications (see Online Appendix C). Values expressed in euros over 2012.

Figure B.3: Average standardized outcomes, by LTC purchasing regions (Analyses A, B and C)

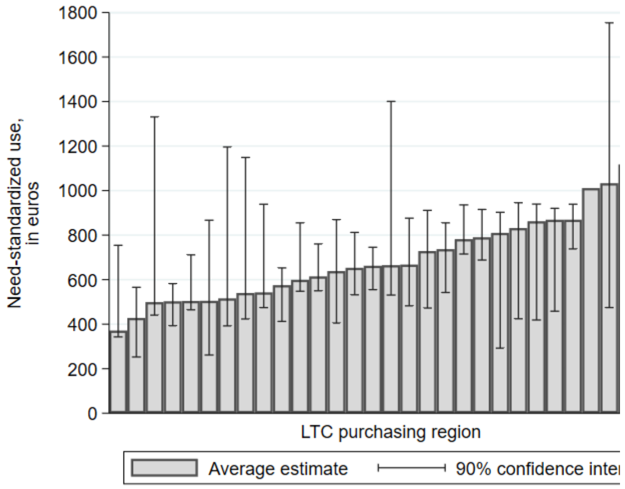

Panel A: Average need-standardized use by LTC purchasing region (Analysis A)

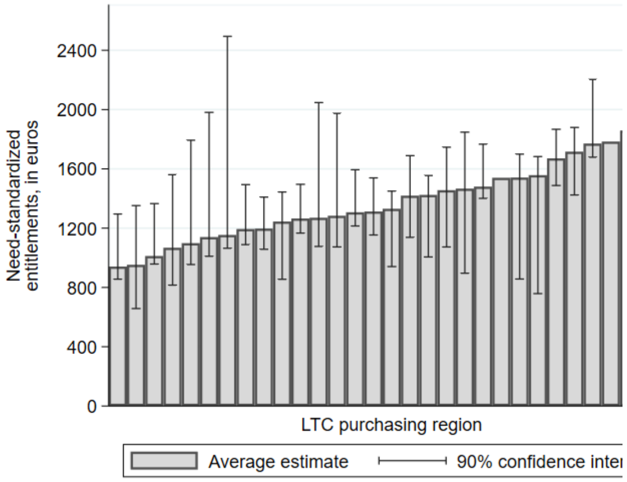

Panel B: Average need-standardized entitlements by LTC purchasing region (Analysis B)

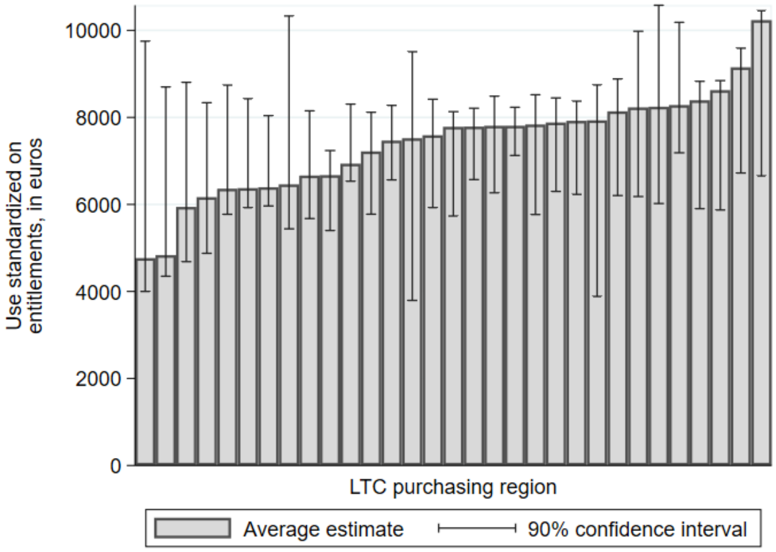

Panel C: Average use standardized for entitlements by LTC purchasing region (Analysis C)

Notes: Need-standardized use and entitlements (Analyses A and B) and use standardized for entitlements (Analysis C) are defined in Equations (3.A) to (3.B). Asymmetric 90%-level confidence interval based on 1,000 Bootstrap replications (see Online Appendix C). Values expressed in euros over 2012. The confidence intervals for region 27 (Panel A) and for region 28 (Panel B) being extremely large, they are not displayed for better readability of the graphs.

Table B.III: Estimates for gender and household composition

	Analysis A		Analysis B		Analysis C	
	(1A)	*(2A)*	*(1B)*	*(2B)*	*(1C)*	*(2C)*
Woman	45.6	190.1*	-29.9	173.1	1073.7***	778.7**
	(79.8)	(104.0)	(119.9)	(154.3)	(241.2)	(371.0)
Partner	-213.3***	-87.5	-384.9	-210.5		-431.5
	(62.2)	(69.2)	(236.0)	(253.9)		(741.3)
Woman x Partner		-213.3***		-304.0**		-167.9
		(77.6)		(133.0)		(550.8)
Number of household members	-99.1*	-85.0	20.8	37.1		-1071.8***
	(52.5)	(53.8)	(213.8)	(214.4)		(391.7)
N	154,646	154,646	154,646	154,646	14,136	14,136

Notes: *** $p < 0.01$, ** $p < 0.05$, * $p < 0.10$. Standard errors in brackets. Estimates from the regression of home care use (Analysis A) or home care entitlements (Analysis B) over 2012 on need variables and non-need factors; estimates from a constrained linear regression of home care use over 2012 on entitlements and non-need factors. In the baseline analysis, gender is a non-need factor but having a partner and number of household members are need variables (Columns (1A) and (1B); there are no need variables in Analysis C); in a complementary analysis, all these 3 variables are treated as non-need factors (Columns (2A), (2B) and (2C)).

Appendix C: Inference on concentration and horizontal inequity indexes

C.1. Standard error of the concentration index of LTC use and LTC entitlements

Inference on a concentration index derived from microdata was derived in Kakwani *et al.* (1997).[7] The standard error of the concentration index of a variable can be obtained using a “convenient regression” and can accommodate survey weights.

For outcome Y, denoting r_i^{SES} is the weighted fractional rank of individual *i* in the distribution of SES, we can derive an estimate of the concentration index by estimating by OLS following regression:

$$Y_i = \theta_0 + \theta_1 r_i^{SES} + \epsilon_i \qquad \text{(C.1.)}$$

The estimate of $CI(Y)$ is given by:

$$\hat{\theta} = \frac{2\sigma_r^2}{\hat{\mu}} . \hat{\theta}_1 \qquad \text{(C.2.)}$$

Where σ_r^2 is the variance of the (weighted) fractional rank and $\hat{\mu}$ is an estimate of the population-average of Y. Given that the weighted OLS predicted value of Y is equal to the population average of Y, and that the mean of the weighted fractional rank is 0.5, we can rewrite:

$$\hat{\theta} = \frac{2\sigma_r^2}{\hat{\theta}_0 + \hat{\theta}_1/2} . \hat{\theta}_1 \qquad \text{(C.3.)}$$

Given that the estimate of $CI(Y)$ has been rewritten as a function of the regression coefficients, a standard error can be derived applying the delta method. In Stata, we use the `nlcom` command, which supports probability weights. In addition, this command allows us to take into account the clustered sampling design of the Dutch Health Monitor survey, by correcting the standard error for within-cluster correlation.[8]

Using this approach, we can derive standard errors associated with the concentration of LTC use in Analysis A, the concentration of LTC needs in Analysis B and the concentration of LTC

[7] We also refer to the manual by O'Donnell *et al.* (2008), which provides an extensive presentation of the formulas for the concentration and horizontal inequity indexes and the computation of their standard errors.
[8]See O'Donnell *et al.* (2008), pp. 103-104, for more details.

use among the elderly eligible for home care in Analysis C. However, in our Tables of results, we have provided confidence intervals derived using an alternative method, i.e. a cluster-bootstrap approach (cf. *infra*). Inference based on the delta method (available on demand) and inference based on the bootstrap approach lead to the same conclusions regarding the statistical significance of the concentration indices of use and entitlements at the conventional thresholds of 1%, 5% and 10% levels.

C.2. Standard error of the concentration of needs, the horizontal inequity index and the contributions of non-need factors

One way of obtaining standard errors for the concentration index of the need-predicted outcome and for the horizontal inequity index is to apply the convenient regression approach described here-above to the need-predicted outcome and the need-standardized outcome respectively, instead of applying to the raw outcome. However, this approach would not take into the fact that there is sampling variability in the need-predicted and need-standardized outcomes for Analyses A and B, as they are derived from the OLS regression predictions. We therefore conduct inference based on a bootstrap procedure.[9]

The standard assumption for a bootstrap procedure is that the observed sample is a random sample of the underlying population and that observations within the sample are independent (van Doorslaer *et al*., 2004). As the Health Monitor survey is based on a two-stage sampling design with unequal sampling probability, the standard assumption does not hold. We therefore implement a cluster bootstrap while taking into account the unequal sampling probability design.

[9] Bootstrap is a resampling method for estimating the sampling variability of sample estimates by randomly drawing multiple samples with replacement from the original sample.

We proceed in three steps:

a. We draw a random subsample b (with replacement) of the 335 primary sampling units (PSUs);[10]
b. In sample b, we adjust the survey weights in the following way: for individual i, we compute the share of the study population it originally represented, $p_i = \frac{w_i}{\sum_{j=1}^{N} w_j}$, where N is the original sample size and w_i the probability weight for individual i. The adjusted probability weight for individual i in sample b is computed as: $\tilde{p}_i = p_i . \frac{\sum_{j=1}^{N^b} w_j}{n_i}$, where N^b is the size of sample b and n_i is the number of times individuals i was drawn in sample b (as the random is drawn with replacement, one cluster and thus one individual may show up several times in a given bootstrap sample). This adjustment ensures that the relative weighting of individuals who are selected in sample b is the same as in the original population.[11]
c. We use sample b to replicate our core analysis: we run the OLS regressions, derive the concentration and horizontal inequity indexes, as well as the contributions of non-need factors, while taking into account the clustered nature of the bootstrap sample and the weights.

We replicate steps 2 to 4 B=1,000 times, so that we obtain B different samples, with B values of our statistics of interest (e.g. concentration index). Finally, for each statistics of interest S, we draw the distribution of its value over the b=1,…, B samples. The upper bound of the 0.5% and of the 99.5% (respectively of the 2.5% and of the 97.5%, and of the 5% and of the 95%) smallest values provide a bootstrapped 99% level (respectively a 95% level and 90% level) confidence interval for statistics S.

Note that the Lasso procedure itself is not bootstrapped, as this would result in excessive computational time. In addition, because of the bootstrap analysis, we have to regroup the LTC

[10] For this (bootstrap) sample b, the sample size N^b depends on the number of observations in the selected clusters.
[11] This approach is yet not ideal as it is not possible to take into account the individuals who are not selected in sample b in the reweighting process. Another possible approach would be to expand the sample by multiplying each observation by its survey weight, such that we obtain a final sample, of size N' where all observations have a probability sampling weight equal to 1. However, given the large sample size and the fairly large dispersion of weights, expansion would result in a dataset too large to be handled with Stata®.

purchasing regions for a technical reason: several of the 32 regions are relatively small (less than 2,500 elderly from our entire study sample, and many less when we focus on the subsample eligible for home care in Analysis C). This implies that some of the bootstrap subsamples do not include any individual from these small regions, and estimates of the coefficients of the LTC purchasing regions cannot be systematically derived.

To circumvent this issue, we have grouped the 32 regions into 8 groups in a data-driven way. We have run a regression of home care use on need variables and the non-need factors (Equation (1.A), Section 4). We have retrieved the estimates of the coefficients of the 32 regions (the coefficient for the reference region being set to 0). We have then ordered the 32 regions from the one with the lowest coefficient to the one with the highest. Finally, we have divided the regions into 8 groups of 4, from the lowest-use regions to the highest-use group.

Table C.I: Grouping of the LTC purchasing regions for the econometric analysis

	**Group 1**	**Group 2**	**Group 3**	**Group 4**
Regions	6, 8, 9 and 32	2,5, 11 and 28	1, 3, 13 and 15	12, 20, 21 and 26
N	*5,304*	*12,969*	*6,203*	*20,528*
	**Group 5**	**Group 6**	**Group 7**	**Group 8**
Regions	10, 23, 24 and 29	7, 14, 25 and 30	4, 16, 18 and 31	17, 19, 22 and 27
N	*29,724*	*27,251*	*29,569*	*23,161*

Notes: The number identifying each region is the one used by Statistics Netherlands.

Appendix D: Deriving and decomposing the horizontal inequity index

The aim of this Appendix is twofold: first, we aim at motivating the 'conservative' approach to the horizontal inequity index for Analyses A and B that we adopt in our fourth robustness check (provided in Online Appendix E). This approach relies on a decomposition of income-related inequalities proposed by Wagstaff et *al.* (2003). Second, we show how the standardization for entitlements that we propose in Analysis C can be made consistent with a decomposition of HI into the contributions of non-need factors, which in turn motivates the estimation of a constrained linear regression analysis when testing for the sources of horizontal inequity at the stage of the conversion of entitlements into care use (Section 5.3 and Table B.II in Online Appendix B).

D.1. Decomposition of HI^A and HI^B

Following Wagstaff et *al.* (2003), we can decompose CI^A into:

$$CI^A = \sum_{k=1}^{K} \frac{\bar{x}^k}{\bar{U}} \hat{\alpha}^k CI(x^k) + \sum_{l=1}^{L} \frac{\bar{z}^l}{\bar{U}} \hat{\alpha}^l CI(z^l) + CGE^A(u) \quad \textit{(D.1.A)}$$

$$= CI(\hat{U}^A) + HI^A$$

Where $\bar{x}^k$ (resp. $\bar{z}^l$) is the population-average of the need factor x^k (resp. non-need factor z^l), and $CI(x^k)$ (resp. $CI(z^l)$) is the concentration index of variable x^k (resp. z^l). $CGE^A(u)$ is called the generalized residual and equals the concentration index of the residual term from the estimation of Equation (1.A), rescaled by $\bar{\hat{u}}/\bar{U}$:

$$CGE^A(u) = 2cov(\hat{u}, r^{SES})/\bar{U} \quad \textit{(5)}$$

HI^A is conventionally defined as: $\sum_{l=1}^{L} \frac{\bar{z}^l}{\bar{U}} \hat{\alpha}^l CI(z^l)) + CGE^A(u)$. A more conservative approach would be to consider that unobserved determinants of LTC access are also (unobserved) need factors. In such a case, the generalized residual in Equation (D.1.A) would be subtracted from HI^A and the 'conservative' horizontal inequity index for Analyses A writes:

$$HI^{cons,A} = \sum_{l=1}^{L} \frac{\bar{z}^l}{\bar{\bar{U}}} \hat{\alpha}^l CI(z^l) \qquad \text{(D.2.A)}$$

while $CI(\hat{U}^A) + CGE^A$ measures the concentration of both observed and unobserved needs across the income distribution. [12]

Similarly, for Analysis B, we can write:

$$CI^B = \sum_{k=1}^{K} \frac{\bar{x}^k}{\bar{\bar{E}}} \hat{\beta}^k CI(x^k) + \sum_{l=1}^{L} \frac{\bar{z}^l}{\bar{\bar{E}}} \hat{\beta}^l CI(z^l) + CGE^B(\varepsilon) \qquad \text{(D.1.B)}$$

$$= CI(\hat{U}^B) + HI^B$$

$$HI^{cons,B} = \sum_{l=1}^{L} \frac{\bar{z}^l}{\bar{\bar{E}}} \hat{\beta}^l CI(z^l) \qquad \text{(D.2.B)}$$

The estimates of $HI^{cons,A}$ and $HI^{cons,B}$ are provided as a robustness check (‘Check 4’) in Online appendix E, Table E.I.

D.2. Decomposition of HI^C

The derivation of HI^C is different from what we do in Analyses A and B, to the extent that in Analysis C use is standardized on a readily available measure of entitlements (we do not need to rely on a regression-based derivation of needs). Still, we can use a similar framework to decompose HI^C into the contribution of non-need factors; in addition, a regression can be used to test for any partial correlation between a non-need factor and home care use, when entitlements are controlled

[12] According to Wagstaff et *al.* (2003), $\frac{\bar{z}^l}{\bar{U}} \hat{\alpha}^l CI(z^l)$ can be thought as the (non-causal) contribution of non-need factor z^l to income-related inequality in care use; as such, HI can be expressed as the sum of the contributions of non-need factors plus the generalized residual. Our empirical analysis does not rely this interpretation, which has been discussed by several recent developments of the equity literature (see Erreygers & Kessels (2013) and Heckley *et al.* (2016)). Whatever the interpretation of $\sum_{l=1}^{L} \frac{\bar{z}^l}{\bar{U}} \hat{\alpha}^l CI(z^l)$, it remains true that the horizontal inequity index, in its conventional definition, equals this term plus the (rescaled) concentration index of the residuals.

for. Regression estimates can then be used to better understand what drives the differences in care use standardized for entitlements across population groups (i.e. horizontal inequity) that we report in Section 5.3.

For Analysis C, we estimate the following constrained linear regression on the subsample of the elderly eligible for some home care:

$$U_i = (\bar{U}^{elig}/\bar{E}^{elig})E_i + Z_i'\gamma^Z + v_i \qquad (1.C)$$

The coefficient of entitlements is constrained to be equal to the ratio of population averages $(\bar{U}^{elig}/\bar{E}^{elig})$. This ensures that we use the vector of estimates of parameters γ^Z and residuals v to decompose income-related inequality in care use as:

$$CI^C = \left(\frac{\bar{U}^{elig}}{\bar{E}^{elig}}\right).\left(\frac{\bar{E}^{elig}}{\bar{U}^{elig}}\right).CI(E)^{elig} + \sum_{l=1}^{L}\frac{\bar{z}^{l,elig}}{\bar{E}^{elig}}\hat{\gamma}^l CI(z^l) + CGE^C(v) \qquad (D.C)$$

$$= CI(E)^{elig} + HI^C$$

Where $\bar{z}^{j,elig}$ is the average of non-need factor z^j in the population eligible for home care. Consistently, HI^C is simply equal to the difference between the concentration index of use and the concentration index of entitlements among the individuals eligible for home care $(CI(U)^{elig} - CI(E)^{elig})$.

In the case of Analysis C, there is no conservative approach to inequity analogous to the one considered for Analyses A and B: when assessing inequity in the conversion of entitlements into actual care use, we standardize use for entitlements, which are perfectly observed. In this setting, the error term v necessarily captures unobserved non-need factors, and $CGE^C(v)$ reflects the concentration of these unobserved *illegitimate* determinants of home care use.

Appendix E: Additional robustness checks

In the main text, we have included two robustness checks for the baseline estimates income-related inequity. This Appendix provides four additional robustness checks, whose results are displayed in Table E.I (‘Check 3’ to ‘Check 6’). For better readability, we have reproduced the estimates from the baseline analysis and the first two robustness checks.

[Table E.I. on the following page]

As a third robustness check, we use the Lasso to select *both* the need variables and the non-need factors to be included in the regressions. On the one hand, including as control variables as many non-need factors as available reduces the risk that the correlation between need variables and income rank (which defines fair income-related inequalities in access to care) actually captures a correlation between the income rank and the non-need factors, in the case the latter are correlated with need variables. On the other hand, not submitting the non-need factors to the Lasso selection procedure may lead to exclude some need variables that are highly correlated with some non-need factors. This might distort our estimates of legitimate needs and of their distribution across income levels. We have replicated Analyses A and B using the Lasso to statistically select simultaneously a subset of need variables and a subset of non-need factors. The point estimates for HI (Table E.I, ‘Check 3’) are very close to our baseline estimates.

Table E.I: CI and HI for Analyses A and B – additional robustness checks

Analysis:	*Variant*	*CI(outcome)*<br>(1)	*HI(outcome)*<br>(2)
A. LTC use (equity overall)	Baseline		-0.036***<br>[-0.057;-0.010]
	Check 1: having a partner is a non-need factor		-0.058***<br>[-0.066;-0.017]
	Check 2: no administrative data on health care use	-0.340***<br>[-0.361;-0.315]	-0.043***<br>[-0.077;-0.035]
	Check 3: both need variables and non-need factors are selected with Lasso		-0.029***<br>[-0.044;-0.011]
	Check 4: unobserved factors correlating with income are need variables		-0.037***<br>[-0.059;-0.011]
	Check 5: OECD modified equivalence scale & equal sharing within couples	-0.313***<br>[-0.335;-0.288]	-0.035***<br>[-0.055;-0.009]
	Check 6: OECD square root scale & sharing rule "63% to women"	0.020**<br>[0.004;0.040]	0.001$^{n.s}$<br>[-0.016;0.016]
B. LTC entitlements (equity at eligibility stage)	Baseline		-0.016$^{n.s.}$<br>[-0.036;0.005]
	Check 1: having a partner is a non-need factor		-0.025*<br>[-0.041;0.000]
	Check 2: no administrative data on health care use	-0.288***<br>[-0.311;-0.267]	-0.020**<br>[-0.045;-0.005]
	Check 3: both need variables and non-need factors are selected with Lasso		-0.011$^{n.s}$<br>[-0.026;0.004]
	Check 4: unobserved factors correlating with income are need variables		-0.018$^{n.s}$<br>[-0.038;0.004]
	Check 5: OECD modified equivalence scale & equal sharing within couples	-0.268***<br>[-0.291;-0.247]	-0.015$^{n.s}$<br>[-0.034;0.006]
	Check 6: OECD square root scale & sharing rule "63% to women"	0.002$^{n.s.}$<br>[-0.017;0.022]	-0.000$^{n.s}$<br>[-0.014;0.013]
C. LTC use (equity at use stage)	Baseline	-0.095***<br>[-0.113;-0.071]	-0.054***<br>[-0.065;-0.040]
	Check 5: OECD modified equivalence scale & equal sharing within couples	-0.086***<br>[-0.104;-0.061]	-0.046***<br>[-0.057;-0.033]
	Check 6: OECD square root scale & sharing rule "63% to women"	-0.015*<br>[-0.029;0.022]	0.015**<br>[0.002;0.031]

Notes: Standard errors in brackets, computed using the convenient formula approach supporting survey weights (O'Donnell *et al.*, 2008); 95% confidence intervals in parentheses; a cluster-bootstrap approach (1,000 replications) is used to construct confidence intervals (see Online Appendix C).
*** $p < 0.01$, ** $p < 0.05$, * $p < 0.10$, $^{n.s.}$ $p \geq 0.10$.

Getting to our fourth robustness check, our baseline analysis relies on the - standard - assumption that the unobserved determinants correlating with the income rank are *illegitimate* determinants of care use. But what if, in spite of our extensive set of health-related information, we fail to observe some (pro-poor distributed) dimensions of needs that are known to CIZ assessors? In particular, an adapted house can improve the capacity of the elderly with functional limitations to perform the activities of daily living without human assistance (Hoenig *et al*., 2003); a more accessible dwelling can also delay nursing home entry (Diepstraten *et al*., 2020). If lower-income elderly are less likely to have adapted houses – which we do not know from our data but is suggested by Diepstraten *et al*. (2020) -, their legitimate care needs would be higher than our estimates indicate. To check that the finding of no pro-rich horizontal inequity in home care access is not driven by our implicit assumption that the unobserved factors co-determining use are *need-related*, in a third robustness check we relax this assumption. With this more ‘conservative' approach (Bago d'Uva *et al*., 2009), estimates point to an absence of income-related horizontal inequity at both the eligibility stage and overall: HI is now extremely close to zero for both Analyses A and B.[13]

Our analysis of income-related inequity in care use relies on the assumptions that: (i) the OECD square root equivalence scale correctly reflects economies of scale for (household-level) expenditures, and (ii) economic resources are shared equally in the household (between spouses).

If (i) is incorrect, the ranking in the income distribution of singles relative to that of the elderly with a spouse would be incorrect. If (ii) is untrue, then the ranking of men with a spouse relative to that of women with a spouse is incorrect. Given that the share of singles (mostly widows) is higher among women, a sharing rule of household resources unfavorable to women would also imply that relatively more women are over-ranked in the distribution of income, relative to men. As both spousal status and gender exhibit practically and statistically significant partial correlation with home care use and/or entitlements (holding other needs and non-need factors fixed), violation of (i) and/or (ii) could bias our assessment of income-related inequity in care use.

[13] This robustness check is based on the decomposition formula provided in Wagstaff *et al.* (2003); it amounts to computing HI as the difference between CI and the sum of the concentration of need variables and the generalized residual (formula provided in Online Appendix D.1).

Estimates of economies of scale and sharing rules in couples are available for the Dutch 65+ population. Using consumption survey data, Cherchye *et al.* (2012) estimate an economies of scale parameter of 0.32. They also estimate a sharing rule that is *more* beneficial to women (average of 0.63 and standard deviation of 0.03 among couples).

The OECD-square root equivalence scale we use in our baseline analysis reflects an economies of scale parameter of 0.41 for a two-person household. The OECD modified scale, which was standardly used until the 2000s, reflects an economies of scale parameter of 0.33. We therefore run two additional robustness checks: first, we re-compute equivalized income using the OECD modified scale; second, we assume that women in a couple receive 63% of the household income, after economies of scale have been taken into account.

Changing the equivalence scale is innocuous; however, assigning more resources to women somewhat affects the results. Like in the baseline analysis, there is no evidence of socio-economic inequity in entitlements for home care; but the pro-poor gradient in the conversion of entitlements into use fades out and even becomes slightly pro-rich. This is consistent with the fact that women were found to convert more of their entitlements on average, and robustness check #6 makes women relatively richer. This scenario may over-estimate the pro-richness of the distribution of home care use, for given entitlements. The intra-household sharing rule estimated by Cherchye *et al.* (2012) used data from 1978 to 2004 and their results suggest that resource sharing became more equal over the period. Re-allocating 63% of the household resources to women among the 65+ population in 2012 may thus make women with a partner artificially richer, relative to men with a partner and single individuals. Furthermore, Cherchye *et al.* (2012) find substantial heterogeneity in the sharing rule across households. We thus take the results from ‘Check 6’ with caution.

Appendix F: List of datasets used

This research relies on data from Statistics Netherlands (CBS) and the Municipal Health Services (GGDs). It has been registered in the Gezondheids monitors repository as part of project 'Financial and health risks: household decisions and government intervention'.

To facilitate the reproducibility of our results, we provide in Table F.I the list of the datasets that we linked together, together with their name in the CBS environment and the version that was used.

Table F.I: List of datasets used in the analysis

Dataset	Content	Version
GEMON	Health Monitor survey (wave 2012)	2012
GBAPERSOONTAB	Population registers (age, gender and migrant background)	2017 – V1
GBAOVERLIJDENTAB	Death records	2017 – V1
GBAHUISHOUDENSBUS	Household composition	2017 – V1
GBAVERBINTENIS-PARTNERBUS	Formal unions	2014 – V1
GBAADRESOBJECTBUS	Address identifiers (main)	2017 – V1
VSLGWTAB	Location of addresses (main)	
NIETVSLGWTAB	Location of addresses (complementary)	2018 – V4
KINDOUDERTAB		2012 – V1
INDICAWBZTAB	CIZ eligibility indications	2012 – V1
GEBZZVTAB	Use of AWVZ-funded home care	2012 – V2
ZORGMVTAB	Use of AWBZ-funded institutional care	2012 – V1
PGBAWBZTAB	Use of AWVZ-funded LTC vouchers	2012 – V1
MEDICIJNTAB	Drug use	2011 – V1
ZVWZORGKOSTENTAB	Health care costs	2011 – V1
Integraal Huishoudens Inkomen	Household income (tailor-made from dataset IHI)	2012
Integraal Vermogen	Household wealth (tailor-made from dataset VEHTAB)	2012

Notes: The description of these datasets is provided by Statistics Netherlands at https://www.cbs.nl/en-gb/our-services/customised-services-microdata/microdata-conducting-your-own-research/microdata-catalogue.

To link each municipality to one of the CIZ regional offices and to one of the LTC purchasing regions, we also used the table of correspondence "GIN - Gebieden in Nederland" (2013-V1).

Finally, we link each individual with a migrant background (first or second generation migrant) to her or his land of origin using a dedicated table of correspondence.[14] Each land is classified by Statistics Netherlands into one of 7 categories: (1) the Netherlands, (2) Suriname, (3) the so-called Dutch Caribbean, (4) Morocco, (5) Turkey, (7) Western countries other than the Netherlands and (7) other non-Western countries. Categories (4) and (5) correspond to the countries from which the highest proportion of individuals living in the Netherlands but with a migrant background come from; categories (2) and (3) encompass countries that used to be Dutch colonies, with the exception of Indonesia (which is classified by Statistics Netherlands in the category of the Western countries). In our empirical analysis, we group together categories (2), (3), (4), (5) and (7).

[14] File “120123 omrekentabel land naar herkomstgroepering.xls” in the CBS remote environment, folder “metadata/Utilities/Code_Listings/Landen- en nationaliteitscodes”.

Additional References

This list provides the references mentioned in the Supplementary Material and not provided in the list of references of the main text.

Bago d'Uva, T.M., Jones, A.M. & van Doorslaer, E.K.A. (2009). Measurement of Horizontal Inequity in Health Care Utilisation using European Panel Data. *Journal of Health Economics*, *28*(2), 280-289.

Belloni, A., Chen, D., Chernozhukov, V., & Hansen, C. (2012). Sparse models and methods for optimal instruments with an application to eminent domain. *Econometrica*, *80*(6), 2369-2429.

Diepstraten, M., Douven, R., & Wouterse, B. (2020). Can your house keep you out of a nursing home?. *Health Economics*, *29*(5), 540-553.

van Doorslaer, E. , Koolman, X., & Jones, A. M. (2004). Explaining income-related inequalities in doctor utilisation in Europe. *Health economics*, *13*(7), 629-647.

Erreygers, G., & Kessels, R. (2013). Regression-based decompositions of rank-dependent indicators of socioeconomic inequality of health. In

Heckley, G., Gerdtham, U. G., & Kjellsson, G. (2016). A general method for decomposing the causes of socioeconomic inequality in health. *Journal of health economics*, *48*, 89-106.

Hoenig, H., Taylor, D. H. Jr., & Sloan, F. A. (2003). Does assistive technology substitute for personal assistance among the disabled elderly? American Journal of Public Health, 93(2), 330–337.

Kakwani, N., Wagstaff, A., & van Doorslaer, E. (1997). Socioeconomic inequalities in health: measurement, computation, and statistical inference. *Journal of Econometrics*, *77*(1), 87-103.

Tibshirani R. (1996). Regression shrinkage and selection via the Lasso. *Journal of the Royal Statistical Society Series B (Methodological)* 58 (1): 267-288.

Wagstaff, A. & van Doorslaer, E. & Watanabe, N. (2003). On decomposing the causes of health sector inequalities with an application to malnutrition inequalities in Vietnam, *Journal of Econometrics*, 112(1), pp. 207-223.
